# Sources of hydrocarbons and their risk assessment in seawater and sediment samples collected from the Nile Delta coast of the Mediterranean Sea

**DOI:** 10.1038/s41598-024-55339-4

**Published:** 2024-03-01

**Authors:** Mohamed A. Hassaan, Safaa Ragab, Amany El Sikaily, Ahmed El Nemr

**Affiliations:** Environment Division, National Institute of Oceanography and Fisheries (NIOF), Kayet Bey, P.O. 21556, Elanfoushy, Alexandria Egypt

**Keywords:** PAHs, *n*-alkanes, Pollutants source identification, Seawater and sediment, Mediterranean, Risk assessment, Environmental monitoring, Risk factors, Environmental chemistry

## Abstract

The aim of this work is to examine the levels, distribution, bases, and hazards of *n*-alkanes (*n*-C9 to *n*-C20) and PAHs in the seawater and sediments around oil production locations in the whole delta region. The variations in the levels of PAHs and *n*-alkanes in seawater and sediment of the Nile delta coast of the Mediterranean were investigated using GC–MS/MS. The Σ*n*-alkanes residues ranged between 12.05 and 93.51 mg/L (mean: 50.45 ± 17.49 mg/L) and 4.70 to 84.03 µg/g (mean: 31.02 ± 27.995 µg/g) in seawater and sediments, respectively. Total PAHs concentrations ranged between 4.485 and 16.337 μg/L (average: 9.47 ± 3.69 μg/L) and 1.32 to 28.38 ng/g (average 8.61 ± 7.57 ng/g) in seawater and sediment samples, respectively. The CPI (carbon preference index) values fluctuated between 0.62 and 1.72 (seawater) and from 0.234 to 2.175 (sediment), proposing the variation sources of *n*-alkane in the studied area. PAHs concentrations were lower than the Effective Range Low (ERL) and Effective Range Median (ERM) levels. The Toxic Equivalent Quotient (TEQ) values oscillated between 0.002 and 6.84 ng/L and from 3.72 to 13.48 ng/g for the seawater and sediment samples, respectively. The Ant/(Ant + Phe) ratio in sediment and seawater samples indicated a pyrolytic source while the BaA/(BaA + Chry) ratio indicates petrogenic sources in most of the studied stations.

## Introduction

The widespread nature of *n*-alkanes and PAHs resulting from their extensive usage as raw materials for industries and energy sources is connected to their environmental predominance^1–3^. According to El Nemr^4^, and Frena et al.^5^, biogenic, pyrolytic, petrogenic and diagenetic processes are potential sources of environmental hydrocarbons. While PAHs petrogenic sources include offshore exploration and oil spills, pyrogenic sources of PAHs include the incomplete combustion of carbonaceous matter, transportation of petroleum, naturally occurring oil seeps, discharges from natural fires, vehicular emissions, and industrial processes^4^.

For decades, the Nile Delta has been one of the most productive areas in Egypt. The region witnessed intense offering of international bids for oil and gas exploration activities, which received expected turnouts from international Oil Companies (IOCs) such as Dana Gas, ENI, BP, SDX and others^6^. Hence, these IOCs attained concession rights in several offshore blocks and have succeeded in making new discoveries. Moreover, the crude oil extracted from the fields of the Gulf of Suez is transported from Cairo to refineries in Tanta and Mostorod, then to Alexandria through two pipelines, Mostorod-Tanta pipeline and Tanta-Alexandria pipeline. Finally, The Nile Delta has eight natural gas pipelines, four of which spread across the region while four exist within it; two pipelines of the latter mainly connect Abu Madi field with Talkha distribution station. The Abu Madi-Talkha pipelines either directly provide the consumers in Talkha with natural gas or further extend to feed electric stations with natural gas in Cairo through the Talkha- Tanta-Cairo pipeline^6^.

Saturated and straight carbon chains with even and odd carbon numbers, which represent anthropogenic and natural sources of hydrocarbon, make up *n*-alkanes. Guidelines were provided by the United Nations Environmental Programme (UNEP) to distinguish between safe (< 10 µg/g) and dangerous (> 10 µg/g) levels for the marine sediment containing *n*-alkanes^7,8^.

According to Sverdrup et al.^9^, El Nemr^7^, Pradhap et al.^10^, and others, the majority of human activities that produce PAHs include incomplete combustion of fossil fuels and biomass and automobile emissions. Khairy et al.^11^ recognize them as poisonous, carcinogenic, mutagenic, bio-accumulative, and persistent. Marine sediments are the last sink for PAHs that are released into rivers and the atmosphere^12^. PAHs may easily interact with suspended objects, sink to the bottom of the ocean, and have a low solubility due to their hydrophobic character. After PAHs attach to sediments, photochemical degradation or microbial oxidation decomposition is frequently weak, causing them to accumulate on sediments^13,14^. As a result, the PAH concentrations in sediments can reflect seawater contamination over an extended period^15^. Water exchange capacity governed how concentrated Total PAHs in the water were distributed in space. The organic matter concentration and sediment texture significantly impacted the geographical scattering of ΣPAHs in the sediment.

*n*-Alkanes and PAHs have been used to study the characterization of organic matter from diverse environmental matrices, including water, suspended particulate matter, and sediments. Some characteristics of *n*-alkanes, such as the Carbon Preference Index (CPI), the mean carbon number (MCN), and the prevalence of an even or odd carbon number for *n*-alkanes, were estimated following^16–18^. These indicators can be used to differentiate between anthropogenic and natural bases of hydrocarbons in the surroundings^19,20^.

For PAHs in both water and sediment; ΣPAHs, ΣLMW, ΣHMW, ΣLMW/ΣHMW Total TEQ, ΣCOMB, ΣCARC, %ΣCOMB/PPAHs, %ΣCARC/PAHs, Nap/Phe, Phe/Ant, Ant/(Ant + Phe), Chr/BaA and BaA/(BaA + Chr) were applied for the purpose of basis diagnosis and calculated according to^10,16–18,21–23^.

Due to recent onshore and offshore gas finds, the Nile Delta has emerged as Egypt's most significant and productive petroleum region. According to Halim^24^ and Leila and Moscariello^25^, it mostly produces petrol with a small amount of oil in a few areas. The ecosystem's structure and function have undergone ongoing, dramatic changes due to these activities, which have adversely affected the environment. Therefore, this investigation examines the levels, distribution, sources, and dangers of 13 US EPA PAHs and *n*-alkanes (oscillating from nonane, *n*-C9, to icosane, *n*-C20) in the seawater and sediments around oil production facilities, which includes the whole delta region (Table S1 in the supplementary data). It is considered that this baseline study is the first to examine and estimate the current distribution of aliphatic and aromatic hydrocarbon load in these three sectors of the Nile Delta's coastal and noncoastal sediments and water.

## Materials and methods

### Materials

The 13 EPA PAHs analyzed in this work were reported in Table S2 (supplementary data), which also summarized some features of the detected PAHs, such as ERL, ERM, TEQ and carcinogenicity data. GC grade *n*-hexane (99.9% pure), CH_2_Cl_2_, and Na_2_SO_4_ anhydrous were all acquired from Merck Millipore. The *n*-alkanes standard solution (analytical standard, contains C9-C20, ~ 40 mg/L each, in hexane, 04070-5ML) from Supelco-solution, Sigma-Aldrich, Germany, was used for their residue computation. The CH_3_OH, CHCl_3_ and acetone were obtained from Sigma Aldrich, Germany. The authorized multi-component PAHs standards combination (EPA 525 PAH Mix A in CH_2_Cl_2_) was provided by Supelco, USA. Hexane was used to dilute the primary PAHs standards, which was the calibration standards.

### Sample collection

**Figure 1:**
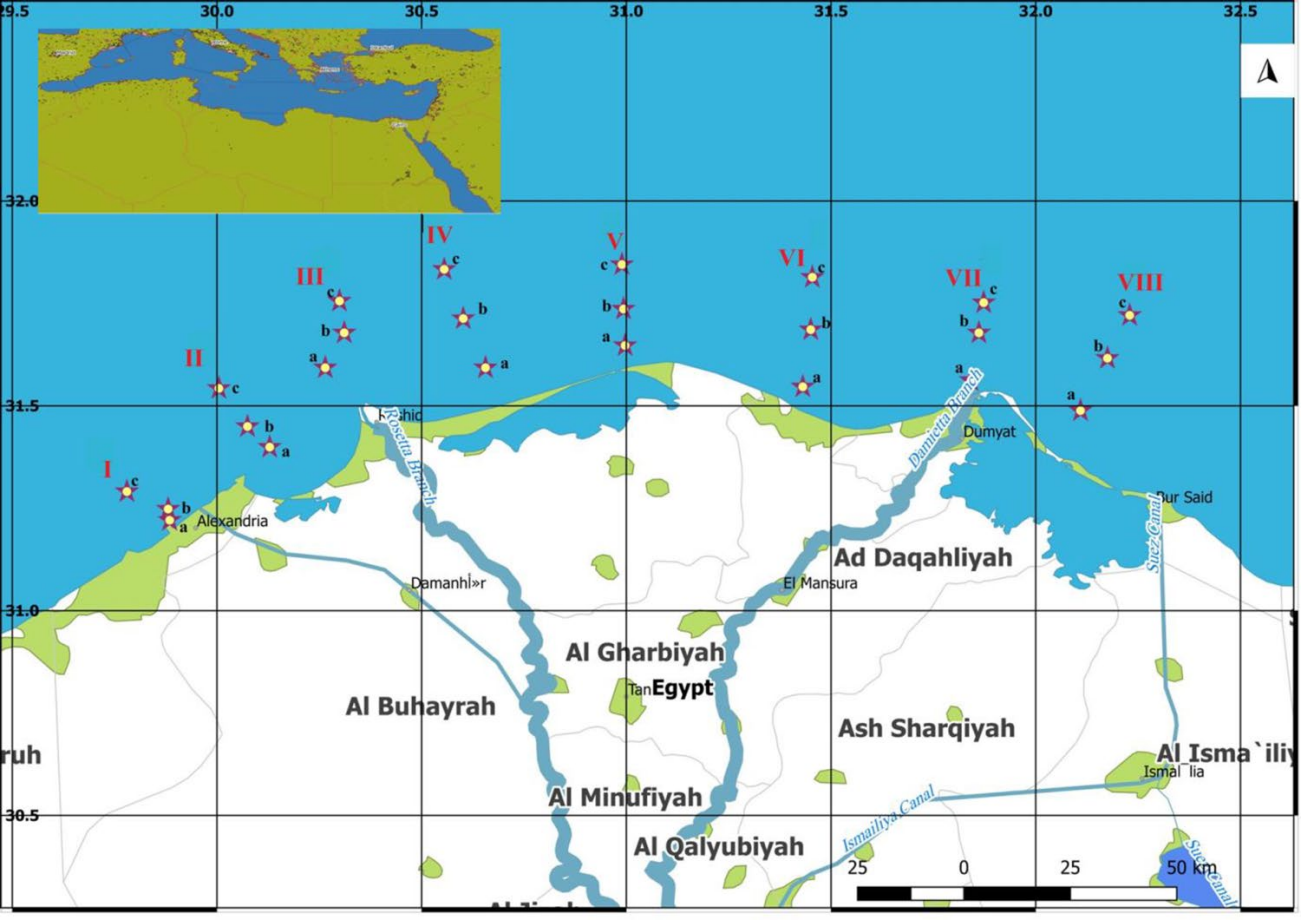
The Nile Delta coastal region is situated in the middle of Egypt's Mediterranean coast between Abu Qir Bay (Alexandria city) in the west (31,022′N-30,018′E) and Manzala Lagoon (Port Said city) outflow in the east (31,017′N-32,012′E) (Software QGIS 3.18; https://www.filehorse.com/download-qgis/61739/)^7,26^.

All samples of seawater and surface sediment were taken from the Nile Delta of the Mediterranean (Fig. 1 and Table S1 in the supplementary data). All samples were taken in the winter of 2020 (December). Standard Niskin Bottles were applied to collect the seawater samples, and a stainless steel grab was used to obtain the silt samples. Two-liter brown glass bottles were used to store a total of 24 surface seawater samples, which were then sealed, kept in the dark, and at a low temperature during their travel to the lab. While the depth ranged from 10 to 50 m, surface sediment samples were taken at 23 locations (except station Ia which including a rocky location) along the northern Mediterranean coast, from the area facing Alexandria city to the area facing Port Said city. Six grabs were collected at each site, and the top 3 cm were scooped into spick-and-span glass bottles, transported frozen, and kept at 20 °C until investigation. Each sample was gathered and weighed at a weight of around 5 g in an aluminum dish. The *n*-Alkanes (C9 to C20) and PAHs were analyzed in the samples.

### Sample pre-treatments

Following a dichloromethane-hexane extraction, seawater samples and heterogeneity of sediment in terms of the PAHs distribution were first investigated to look into variations in PAH contents between replicate seawater and sediment samples. Seawater samples (1 L) were extracted using separating funnel^27,28^. Sediment samples were extracted with the use of ultrasound. Individual sediment samples were taken out of the refrigerator, defrosted at ambient temperature for about five hours, and then dried at 50 °C for a whole night before being subjected to chemical treatment. Then, 5.0 g of anhydrous sodium sulphate was well mixed with 5.0 g of each sample. Each sediment sample was used to make duplicates. Next, the sediment sample was sonicated in an ultrasonic bath with *n*-hexane (2 × 100 mL) for 30 min each, and a third extraction with CH_2_Cl_2_ (100 mL) was performed. The three extracts were mixed, desulfurized using activated copper powder, and concentrated to a few milliliters at around 35 °C in a rotary evaporator. Finally, they were concentrated down to approximately 1 mL using a N_2_ gas stream. Cleaning and fractionation were accomplished by putting the 1 mL concentrated extract through a column made of silica and Al_2_O_3_. The column was made ready by packing with 10 g of silica, 10 g of Al_2_O_3_, and then 1.0 g of Na_2_SO_4_ anhydrous. The saturated aliphatic fraction (F1), 1 mL extract was successfully eluted from the column with *n*-hexane (25 mL). Then 70 mL of dichloromethane and *n*-hexane (80:20) were added for the PAH fraction (F2). To prepare F1 and F2 for further chromatographic examination, a gentle stream of pure N_2_ was used to concentrate them. Hexane was used as the injection solvent since it is a nonpolar solvent, which improved fraction separation^29,30^.

### Instrumental analysis

Because of this analytical method's excellent specificity and sensitivity for contaminated soil samples, individual PAHs were identified and quantified using GC–MS/MS. A DB5 ms ultra-inert capillary column, with an internal diameter of 0.25 mm, length of 30 m, and thickness of 0.25 µm, was used to separate the analytes. Splitless mode and a 1 mL injection volume were used for the analysis. Detection of the analytes was performed by employing a Thermo TRACE™ 1300 gas chromatography equipped with PTV mode splitless injector (Temperature 80 °C, 1 min as splitless time, 5 mL/min as a purge flow, 20 mL/min for 5 min as a carrier gas saver flow, Transfer temperature delay 1 min, Injection pressure 70 kPa for 0.1 min, Transfer pressure 210 kPa, 10 °C/sec as transfer rate, 300 °C as transfer temperature, 3 min as transfer time, 10 °C/sec as a cleaning rate, 320 °C as a cleaning temperature, and 10 min as a cleaning time) coupled to a TSQ 8000 Triple Quadrupole Evo Mass Spectrometer (Thermo, USA) working in selected reaction monitoring (SRM) mode and the Ar gas collision cell was set at 1.5 mL/min. Thermo TriPlus RSH Autosampler was used^31–34^.

The transfer line was set to 300 °C, the ion source temperature was set to 270 °C, the total scan duration was set to 0.1 s, the predicted chromatographic peak width was set to 2.0 s, and the timed scan type was set to SRM for the study of PAHs. The integrated peak area ratio of the target ion to the external standard was used to quantify the analytes. Target ions and retention time order allowed for the identification of the PAH analytes. Table S3 (supplementary data) displays the SRM and EI energies applied to PAHs. The GC oven temperature program used for PAH analysis starts at 60 °C and holds that temperature for 1 min before increasing to 140 °C at a ramping of 20.0 °C/min and holding that temperature for 0.0 min, then increase to 300 °C at a ramping of 5.0 °C/min and holding for 4 min.

The transfer line was set to 300 °C, the ion source temperature was set to 270 °C, the total scan duration was set to 0.1 s, the predicted chromatographic peak width was set to 2.0 s, and the timed scan type was set to SRM for the study of *n*-alkanes. The integrated peak area ratio of the target ion to the external standard was used to quantify the *n*-alkanes. Target ions and retention time order were used to identify the *n*-alkanes. For *n*-alkanes, the SRM and EI energies are displayed in Table S4 (supplementary data). The GC oven temperature program used for *n*-alkanes analysis starts at 65 °C and holds that temperature for 2 min before increasing to 250 °C at a ramping of 15.0 °C/min and holding that temperature for 0.0 min, then increase to 300 °C at a ramping of 8.0 °C/min and holding for 2 min. Thermo Scientific Xcalibur was used for data capture, reprocessing, and report production^35^.

Quality assurance (QA) and Quality control (QC) techniques were used to guarantee the correctness and precision of the analytical results. Duplicate extract analysis, blank analysis, and reanalysis of samples with relative percent differences greater than 20% were performed^36,37^. Additionally, standard curves were calibrated daily using reference standards, and calibration levels were checked after every ten extract analyses. The analyte amounts in the blank samples was insignificant or below LOD. TSQ instrument's regression coefficient for the analytes calibration curves varied from 0.975 to 0.997. Each method's results were extremely accurate, with a relative error range of 0.1 to 5.0%, and the findings of each approach were quite accurate. The recovery rates of n-alkanes and PAH residues from water and sediment samples ranged from 93.4 to 108.2%.

### Hydrocarbon indices

#### Carbon preference index (CPI)

The CPI was calculated using the following Eq. (1)^16^.

1 $$CPI=\frac{{\sum (C9-C19)}_{ODD}}{{\sum \left(C10-C20\right)}_{EVEN}}$$

where (*C*i − *C*j)_EVEN_ and (*C*i − *C*j)_ODD_ are the concentrations of the *n*-alkane with an even carbon and with an odd carbon numbers, respectively, over the range *i*–*j*.

#### Mean carbon number (MCN)

Determination of the mean carbon number (MCN) for the same sample also gives an indication of the relative input source. Equation (2) was used to determine the mean carbon number (MCN) of the *n*-alkanes in the seawater and sediment samples (Tables 1):

2 $$MCN=\frac{\sum (i\times {C}_{i})}{(T-{C}_{{\text{S}}})}$$

where *C*_i_ and [*T* − *C*_s_] represent, respectively, the concentration of the *n*-alkane with *i* carbon number and that of the *T* − *C*_s_, respectively.

**Table 1:** The *n*-alkanes Average concentrations in Seawater (mg/L) and Sediment (µg/g) Samples.

Compounds site	Σ*n*-alkanes	Σ(C9–C19)_odd_	Σ(C10–C20)_even_	CPI	MCN	Σ*n*-alkanes	Σ(C9–C19)_odd_	Σ(C10–C20)_even_	CPI	MCN
	Seawater samples					Sediment samples				
Ia	44.23	18.32	25.91	0.71	15.05	N/A	N/A	N/A	N/A	N/A
Ib	40.85	17.16	23.69	0.72	14.72	7.64	4.00	3.65	1.10	15.36
Ic	48.94	19.93	29.01	0.69	14.78	52.07	12.89	39.17	0.33	16.38
IIa	53.96	22.70	31.26	0.73	14.50	84.03	18.13	65.89	0.28	16.46
IIb	46.63	17.84	28.79	0.62	14.94	65.95	13.52	52.43	0.26	16.56
IIc	72.70	36.06	36.64	0.98	15.26	74.86	15.40	59.46	0.26	16.57
IIIa	49.09	21.71	27.38	0.79	14.40	51.49	9.75	41.74	0.23	16.92
IIIb	60.92	32.02	28.89	1.11	15.07	57.60	11.82	45.78	0.26	16.53
IIIc	42.84	18.78	24.07	0.78	14.14	44.91	10.20	34.70	0.29	16.45
IVa	54.56	25.48	29.08	0.88	14.15	6.17	3.26	2.91	1.12	14.24
IVb	34.61	14.63	19.98	0.73	13.72	44.81	8.93	35.89	0.25	16.98
IVc	12.05	4.94	7.11	0.70	13.15	8.12	4.09	4.03	1.02	14.55
Va	25.30	15.99	9.31	1.72	12.82	4.70	2.42	2.28	1.06	5.98
Vb	42.57	19.33	23.24	0.83	15.14	56.39	13.12	43.27	0.30	6.93
Vc	77.04	36.37	40.67	0.89	15.60	7.62	3.92	3.70	1.06	14.89
VIa	33.30	14.20	19.10	0.74	14.62	7.13	3.67	3.46	1.06	14.36
VIb	45.86	20.53	25.33	0.81	14.95	22.71	15.56	7.15	2.18	15.36
VIc	72.17	39.87	32.29	1.24	15.59	5.86	2.90	2.96	0.98	14.22
VIIa	56.35	24.90	31.46	0.79	15.21	5.64	2.95	2.69	1.10	14.18
VIIb	37.71	16.85	20.87	0.81	14.80	5.92	3.10	2.82	1.10	13.71
VIIc	47.33	21.48	25.85	0.83	14.86	5.78	3.07	2.71	1.13	13.99
VIIIa	93.51	45.84	47.67	0.96	15.83	15.28	8.72	6.55	1.33	15.44
VIIIb	53.22	22.65	30.57	0.74	15.55	6.26	2.86	3.41	0.84	14.60
VIIIc	64.95	29.42	35.53	0.83	15.45	72.52	15.73	56.80	0.28	16.42
MIN	12.05	4.94	7.11	0.62	12.82	4.70	2.42	2.28	0.23	5.98
MAX	93.51	45.84	47.67	1.72	15.83	84.03	18.13	65.89	2.18	16.98
Average	50.45	23.21	27.24	0.86	14.76	31.02	8.26	22.76	0.77	14.65
SD	17.49	9.28	8.77	0.23	0.75	28.00	5.33	23.24	0.51	2.80

*N/A* not available.

#### Toxic equivalent (TEQ)

Equation (3) was used to determine each unique PAH's toxic equivalent (TEQ), which represents the potential toxicity.

3 $$TEQ=\sum iCi\times TEFi$$

where *TEFi* is the toxicity factor of distinct fractions, and *C*_i_ is the concentration of a specific PAH component.

For Statistical analysis in the presented study, SPSS Version 20, was utilized for the Hierarchical cluster analysis dendrograms and for correlation analysis for both PAHs and n-alkanes in water and sediments.

## Results and discussion

### *Composition, distribution, and concentration of n*-alkanes* and PAHs in seawater*

**Figure 2:**
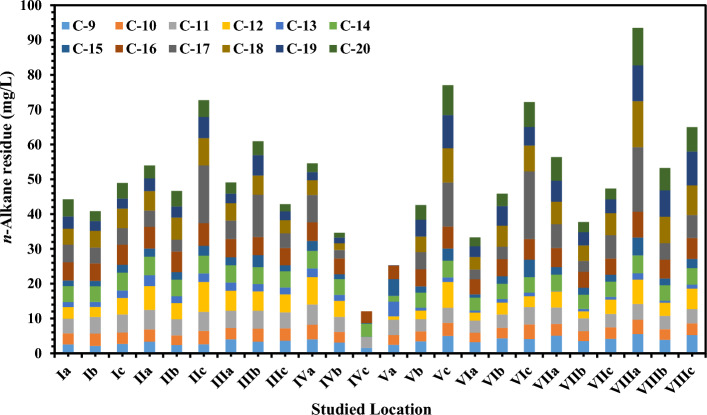
The *n*-Alkanes average concentrations (mg/L) in seawater samples.

**Figure 3:**
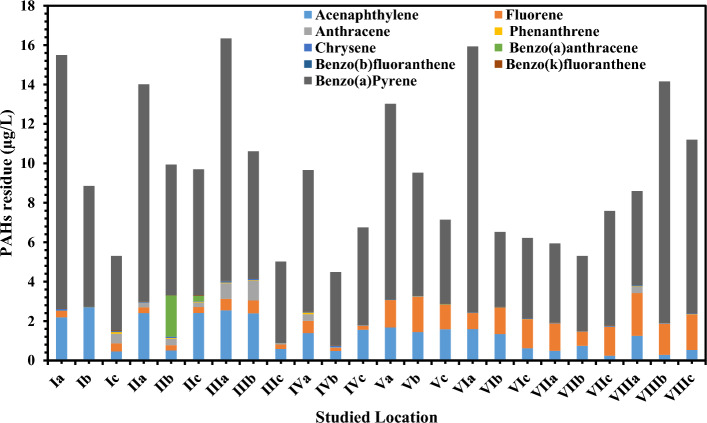
PAHs average concentrations (µg/L) in seawater samples.

In the seawater samples, *n*-alkanes ranged from ND at IVc and Va stations to 19.46 mg/L recorded at VIc (Fig. 2 and Table S5 in supplementary data). The Σ*n*-alkanes ranged from 12.05 mg/L at IVc station to 93.51 mg/L at VIIIa station, with an average 50.45 ± 17.49 mg/L (Table 1). Most stations contain *n*-alkane concentrations extremely higher than the acceptable level given by^38^ and that recorded by the international level of 0.5 mg/L. Individual and total concentrations of 13 priority PAHs, and ΣPAHs in the seawater are given in (Fig. 3 and Table S6 in supplementary data). The ΣPAHs ranged from 4.49 µg/L at IVb station to 16.34 µg/L at IIIa station, with an average value of 9.47 ± 3.69 µg/L. PAHs ranged from ND at all stations to 13.48 µg/L recorded at VIa. The higher ΣPAH concentration is supposed to be related to high PAH emission in this period (Table S6 in supplementary data) due to the primary human-made sources of PAHs including automobile emissions, incomplete combustion of biomass and fossil fuels^39^. The content of -alkanes in the seawater of delta region had obvious spatial differences, and the concentration of n-alkanes in the III, IV, VII and VIII stations decreased with an increasing offshore distance. The concentration of n-alkanes was higher in the water near the shore, especially in Rasheed Nile branch station III and Dameitta Nile branch station VII. This was primarily because the river carried a substantial amount of n-alkanes into the sea.

### Composition, distribution, and concentration of *n*-alkanes and PAHs in sediment

**Figure 4:**
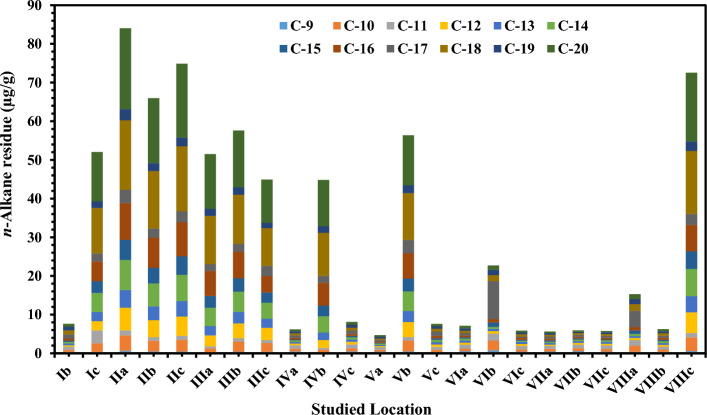
The *n*-alkanes average concentrations in sediment samples (µg/g).

**Figure 5:**
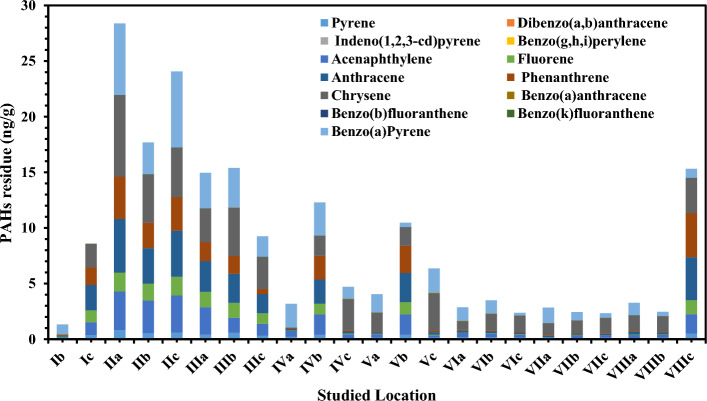
The individual PAHs average concentrations in Sediment samples (ng/g).

In the sediment, *n*-Alkanes ranged from 0.18 µg/g at IIIa site to 20.87 µg/g recorded at IIa site (Fig. 4 and Table S7 in supplementary data). The Σn-alkanes ranged from 4.70 µg/g at Va site to 84.03 µg/g at IIa site, with an average of 31.02 ± 28 µg/g. C-20 recorded the highest average value of 20.87 µg/g, while C9 recorded the lowest average of 0.18 µg/g (Table S7). Individual concentrations of 13 priority PAHs and ΣPAHs in sediment samples are given in Fig. 5 and Tables S8 (supplementary data). The average values ranged from not detected (ND) at most of the stations to 7.34 ng/g at IIa station. The quantities of PAHs in the sediment samples varied widely between the investigated sites. The values oscillated from 1.32 ng/g at Ib station to 28.38 ng/g at IIa station, with an average of 8.61 ± 7.57 ng/g. Chrysene represents the highest concentration in the studied PAHs, while (Da,hA), (InP), (Bg,h,iP), (BbF) and (BkF) represented the lowest concentration in all stations (ND) (Tables S8 in supplementary data). The diverse sources of discharged waters, closeness to human activities, and emissions from fuel burning may be to blame for the variation in PAHs composition along the examined strata (Tables S10 in supplementary data). According to El Nemr^30^ and Beolchini et al.^40^, the chemical makeup of sediments, such as organic matter, clay, and sand, impacts the quantities of PAHs in sediments. Significant PAH readings were characteristic of sediments with significant levels of organic carbon^15,41^. The quantities of 0–100 ng/g indicate light pollution, 100–1000 ng/g indicate moderate pollution, 1000–5000 ng/g indicate high pollution, and higher than 5000 ng/g indicate severe pollution, according to Baumard et al.^42^ grading system for PAH residue in sediments. Light pollution is present in all the sediment samples obtained for the investigation (Fig. 5). The findings revealed that the pollutants were evenly distributed throughout the sediment, with no variation in total PAHs amounts across replicate samples.

### CPI and MCN of n-alkanes

The CPI has been applied to the estimation of the origin of *n*-alkanes. According to Zdanaviciute et al.^43^, the CPI values for petroleum were reported to range from 0.93 to 1.07. However, according to Bi et al.^44^, the CPI values for plants ranged from 2.3 to 54.3. The *n*-alkanes larger contribution from man-made sources, such as petroleum pollution and burning biomass, is therefore indicated by CPI values that are near to unity; higher CPI values imply a bigger contribution from natural sources, such as terrestrial vegetation and biogenic sources^45^. When the carbon preference index is near 1, it is linked to activities like driving and other emissions; when it is more than 1, it is linked to terrestrial vegetation; and when it is lower than 1, it is linked to petroleum^46–48^. Simoneit^49^, also showed that a CPI < 5 has a greater proportion of odd-numbered *n*-alkanes produced by cracking and dehydrating n-alkanes and *n*-alcohols, respectively. The CPI, a measure of biologically produced *n*-alkanes, shows the proportional contribution of *n*-alkanes from natural sources (terrestrial vegetation/biogeneic; high CPI > 1) and man-made sources (biomass burning/petroleum pollution; low CPI < 1) to the total amount of *n*-alkanes^45,50^.

The CPI calculated values for seawater and sediment samples are given in Table 1 and ranged between 0.62 and 1.72 for seawater samples. The values of CPI at IIIb, Va and VIc stations were > 1.0. Thus, the *n*-alkanes at these sites may have come from natural sources, which suggests that terrestrial plant waxes are the main source of *n*-alkanes^51^. The CPI at sites IIIb, IVa, Vc and VIIIa were close to 1.0. Thus the artificial sources could predominantly contribute to the *n*-alkanes, such as vehicle emissions and other activities. Moreover, the CPI at the rest of the sites are < 1 and ranged between 0.62 and 0.83, which may indicate aquatic material such as algae and bacteria^52^. However, there were notable differences in the *n*-alkane compositions at these locations (Table 1).

On the other hand, for the sediment samples, the highest CPI value was found at station VIb with CPI > 2 (Table 1). The CPI values at Ib, IVa, Va, Vc, VIa, VIIa, VIIc and VIIIa locations were > 1.0. Therefore, the n-alkanes at these locations might be derived from natural sources. Vehicle emissions and other activities are linked to CPI values close to 1 in IVc and VIc locations. In this study, the values of CPI larger than 1 demonstrate a preference for *n*-alkanes with odd rather than even carbon numbers. CPI values less than 1 are indicative of the preponderance of even-numbered alkanes. Neither even nor odd numbered alkane preponderance, typical of petroleum and/or mixed sources, is represented by CPI values equal to 1^53^.

For the seawater samples, the MCN values ranged between 12.82 and 15.83 (Table 1). The high MCN values were at the VIIIa site, while the lower ones were at Va with an average value of 14.76 ± 0.75. For sediment, the MCN values ranged between 5.98 and 16.98 (Table 1). The high MCN value was at IVb site, while the lower was at Va site in seawater samples, with an average value of 14.654 ± 2.80. According to Tareq et al.^45^, the low MCN values can be attributed to the trees' high relative abundance compared to grasses and plants during the low flow of organic carbon. In this investigation, the MCN did not substantially correlate with the CPI, indicating that the n-alkane sources at the analyzed locations varied. However, this study's CPI and MCN findings concur with those of^16^.

### Risk assessment, sources and TEQ of PAHs

**Figure 6:**
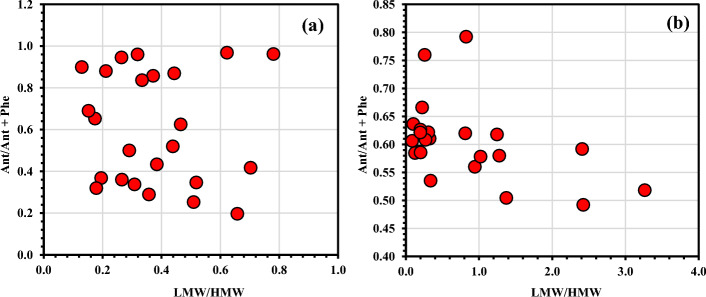
The Ant/Ant + Phe against LMW/HMW ratios cross plots for the potential PAHs source in (**a**) water and (**b**) sediment samples in the studied area.

In Tables 2, 3 and Table S2, for seawater and sediment samples, respectively, the number of rings of PAHs, statistical outputs, toxicity impact range, carcinogenic potency, and molecular weight of the PAHs are displayed. Low molecular weight rings (LMW, 2-rings and 3-rings) and heavy molecular weight rings (HMW, 4-rings and above) were used to categorize the examined PAHs. HMW rings dominated the surface seawater of the Mediterranean's Nile delta and ranged between 56.2 and 88.5% of the ΣPAHs concentration, whereas LMW rings ranged between 11.5 and 43.8% (Table 2). Among the 3 rings PAHs, Acenaphthylene (Ace) were the dominant member compared to others. The LMW/HMW ratio is less than 1 in all the studied seawater samples, which indicates pyrolytic sources (Fig. 6a)^3^. BaP represents the highest concentrations among all the studied HMW PAHs compounds. The source of PAH fractions can be identified by comparing the ratio of low to high molecular weight PAHs^17,18^.

**Table 2:** The Σ PAHs and different ratios of individual PAH in seawater samples (µg/L).

Compounds/stations	ΣLMW (2&3 Rings)	ΣHMW (4 and above)	LMW/HMW	ΣLMW % to ΣPAHs	ΣHMW % to ΣPAHs	TEQ	% ΣCARC/ PAHs	ΣCARC	Phe/Ant	Ant /(Ant + Phe)	Chr/BaA	BaA/(BaA + Chry)
Ia	2.53	12.96	0.20	16.34	83.66	12.87	83.66	12.96	1.72	0.37	22.81	0.04
Ib	2.70	6.16	0.44	30.49	69.51	6.12	69.51	6.16	0.92	0.52	7.85	0.11
Ic	1.44	3.86	0.37	27.12	72.88	3.78	72.88	3.86	0.17	0.86	17.04	0.06
IIa	2.93	11.08	0.26	20.91	79.09	11.01	79.09	11.08	0.06	0.95	25.32	0.04
IIb	1.14	8.80	0.13	11.47	88.53	6.83	88.53	8.80	0.11	0.90	0.03	0.97
IIc	2.98	6.72	0.44	30.74	69.26	6.41	69.26	6.72	0.15	0.87	0.10	0.91
IIIa	3.95	12.39	0.32	24.19	75.81	12.30	75.81	12.39	0.04	0.96	25.45	0.04
IIIb	4.07	6.54	0.62	38.38	61.62	6.46	61.62	6.54	0.03	0.97	17.46	0.05
IIIc	0.88	4.14	0.21	17.46	82.54	4.12	82.54	4.14	0.14	0.88	5.90	0.15
IVa	2.42	7.24	0.33	25.04	74.96	7.19	74.96	7.24	0.20	0.84	12.79	0.07
IVb	0.67	3.82	0.17	14.85	85.15	3.72	85.15	3.82	0.53	0.65	16.09	0.06
IVc	1.78	4.97	0.36	26.35	73.65	4.93	73.66	4.97	2.46	0.29	16.09	0.06
Va	3.07	9.95	0.31	23.59	76.41	9.92	76.41	9.95	1.96	0.34	7.50	0.12
Vb	3.25	6.28	0.52	34.14	65.86	6.25	65.86	6.28	1.89	0.35	5.72	0.15
Vc	2.84	4.31	0.66	39.69	60.31	4.26	60.31	4.31	4.08	0.20	18.06	0.05
VIa	2.42	13.51	0.18	15.17	84.83	13.48	84.83	13.51	2.13	0.32	3.43	0.23
VIb	2.69	3.83	0.70	41.28	58.72	3.80	58.72	3.83	1.40	0.42	10.63	0.09
VIc	2.10	4.12	0.51	33.75	66.25	4.07	66.25	4.12	2.96	0.25	13.38	0.07
VIIa	1.89	4.05	0.47	31.76	68.24	4.00	68.24	4.05	0.60	0.63	12.04	0.08
VIIb	1.47	3.83	0.38	27.77	72.23	3.79	72.23	3.83	1.31	0.43	7.71	0.12
VIIc	1.71	5.88	0.29	22.54	77.46	5.80	77.46	5.88	1.00	0.50	12.17	0.08
VIIIa	3.77	4.83	0.78	43.84	56.16	4.77	56.16	4.83	0.04	0.96	12.25	0.08
VIIIb	1.87	12.29	0.15	13.24	86.76	12.24	86.76	12.29	0.45	0.69	8.26	0.11
VIIIc	2.35	8.85	0.27	21.00	79.00	8.80	79.00	8.85	1.78	0.36	16.45	0.06
MIN	0.67	3.82	0.13	11.47	56.16	3.72	56.16	3.82	0.03	0.20	0.03	0.04
MAX	4.07	13.51	0.78	43.84	88.53	13.48	88.53	13.51	4.08	0.97	25.45	0.97
Average	2.37	7.10	0.38	26.30	73.70	6.95	73.70	7.10	1.09	0.60	12.27	0.16
SD	0.92	3.30	0.18	9.21	9.21	3.28	9.21	3.30	1.10	0.27	7.00	0.25

**Table 3:** The Σ PAHs and different ratios of individual PAH in Sediment samples (µg/L).

Compounds/station	ΣLMW (2&3 Rings)	ΣHMW (4 and above)	LMW/HMW	ΣLMW % to ΣPAHs	ΣHMW % to ΣPAHs	TEQ	% ΣCARC/PAHs	% ΣCOMB/PAHs	ΣCARC	ΣCOMB	Phe/Ant	Ant /(Ant + Phe)	Chry/BaA	BaA /(BaA + Chry)
Ib	0.33	0.99	0.34	25.33	74.67	0.89	68.78	74.67	0.91	0.99	0.87	0.54	0.15	0.87
Ic	6.06	2.52	2.41	70.68	29.32	N/A	24.89	29.32	2.14	2.52	0.69	0.59	1077.03	N/A
IIa	13.78	14.6	0.94	48.56	51.44	6.43	48.52	51.44	13.77	14.6	0.79	0.56	1256.11	N/A
IIb	9.92	7.76	1.28	56.11	43.89	2.85	40.77	43.89	7.21	7.76	0.73	0.58	953.86	N/A
IIc	12.16	11.9	1.02	50.54	49.46	6.84	46.91	49.46	11.29	11.9	0.73	0.58	1540.04	N/A
IIIa	8.30	6.66	1.25	55.5	44.5	3.19	41.62	44.5	6.23	6.66	0.62	0.62	1883.38	N/A
IIIb	6.90	8.49	0.81	44.85	55.15	3.56	51.4	55.15	7.91	8.49	0.61	0.62	246.1	N/A
IIIc	4.18	5.07	0.83	45.22	54.78	1.83	51.23	54.78	4.74	5.07	0.26	0.79	739.41	N/A
IVa	0.78	2.40	0.33	24.58	75.42	2.14	68.63	75.42	2.18	2.4	0.64	0.61	0.09	0.92
IVb	7.11	5.18	1.37	57.88	42.12	2.97	38.97	42.12	4.79	5.18	0.98	0.51	74.17	0.01
IVc	0.72	3.99	0.18	15.28	84.72	1.08	82.64	84.72	3.89	3.99	0.7	0.59	49.52	0.02
Va	0.45	3.60	0.13	11.22	88.78	1.64	85.06	88.78	3.45	3.6	0.71	0.59	59.24	0.02
Vb	8.02	2.45	3.27	76.56	23.44	0.4	19.74	23.44	2.07	2.45	0.93	0.52	440.03	N/A
Vc	0.60	5.77	0.10	9.35	90.65	2.21	88.27	90.65	5.62	5.77	0.57	0.64	49.88	0.02
VIa	0.68	2.2	0.31	23.56	76.44	1.23	70.25	76.44	2.02	2.2	0.61	0.62	15.94	0.06
VIb	0.58	2.92	0.2	16.68	83.33	1.19	78.35	83.33	2.74	2.92	0.6	0.63	42.24	0.02
VIc	0.50	1.86	0.27	21.28	78.72	0.24	73.09	78.72	1.73	1.86	0.64	0.61	47.73	0.02
VIIa	0.23	2.62	0.09	8.05	91.95	1.4	87.7	91.95	2.5	2.62	0.65	0.61	49.56	0.02
VIIb	0.41	2.04	0.20	16.58	83.42	0.75	79.21	83.42	1.94	2.04	0.61	0.62	57.91	0.02
VIIc	0.39	1.94	0.20	16.91	83.09	0.41	77.11	83.09	1.8	1.94	0.71	0.59	39.67	0.03
VIIIa	0.67	2.60	0.26	20.56	79.44	1.11	77.22	79.44	2.53	2.6	0.32	0.76	50.19	0.02
VIIIb	0.45	2.02	0.22	18.28	81.72	0.39	75.41	81.72	1.86	2.02	0.5	0.67	56.2	0.02
VIIIc	10.85	4.47	2.43	70.8	29.2	0.81	25.97	29.2	3.98	4.47	1.03	0.49	560.01	N/A
MIN	0.23	0.99	0.09	8.05	23.44	0	19.74	23.44	0.91	0.99	0.26	0.49	0.09	N/A
MAX	13.78	14.59	3.27	76.56	91.95	6.84	88.27	91.95	13.77	14.59	1.03	0.79	1883.38	0.92
Average	4.09	4.52	0.8	34.97	65.03	1.89	60.94	65.03	4.23	4.52	0.67	0.6	403.85	0.09
SD	4.57	3.42	0.87	22.00	22.00	1.79	21.6	22.00	3.24	3.42	0.18	0.07	562.51	0.25

*N/A* not available.

According to USEPA^21^, the *TEF* of BaA, Chry, BbF, BkF, BaP, InP, and Da,hA are 0.1, 0.001, 0.1, 0.01, 1, and 1, respectively. In this study, the concentrations of InP and Da,hA are not detected so the TEQ is calculated for the rest of the PAH fraction. In the seawater, the ΣTEQ is ranged between 3.7 and 13.5 mg/L (Table 2).

In seawater and sediment, CARC-PAHs (BaA; Chry; BaP; BbF; BkF; Ba,hA; Bg,h,iP; and InP) are eight PAHs typically considered possible carcinogens^54^. BaP is the highly carcinogenic PAHs^55^ and represents the highest concentrations among all the studied PAHs in seawater. The greatest carcinogenic PAH percentage (% CARC = 88.53%) and highest PAHCARC concentration were both found at the Ia station. The concentration of 13.77 µg/L was the highest value of the PAH_CARC_ in seawater samples (Table 2).

The proportion of the total amount of the 13 EPA-PAHs to the total amount of the main combustion-specific compounds (COMB = BbF; BaA; Chry; Pyr; Flu; Bg,h,iP; BaP; InP; and BkF) was studied and reported in Table 2. In seawater samples, the ratio of % ΣCOMB/PPAHs and % ΣCARC/PAHs is the same; also, the % ΣCARC/PAHs is the same as % ΣHMW to ΣPAHs because Pyr, Da,hA, InP, Flu and Bg,hi,P were not detected in all samples collected from the studied locations.

The PAH concentration ratios of the same molecular weight, such as BaA/(BaA + Chry) and Ant/(Ant + Phe), were employed to investigate potential sources of PAHs. The molecular ratios of PAHs in the seawater at several sample locations in this investigation are displayed in Table 2. Ant/(Ant + Phe) ratios were generally higher than 0.1 in all the examined locations, with an average of 0.60. Maciel et al.^56^ proposed two classes of molecular-based techniques to identify the origin of PAH fractions: (i) Pyrolytic sources are indicated by a ratio of Ant/(Ant + Phe) more than 0.1, whereas petrogenic sources are indicated by a ratio less than 0.1. (ii) Pyrolytic sources are indicated by a Flu/(Flu + Pyr) ratio larger than 0.5, whereas petrogenic sources are indicated by a ratio < 0.5. Therefore, the Ant/(Ant + Phe) is larger than 0.1 in our analysis, which points to a pyrolytic source (Fig. 6b).

Additionally, it was shown from the distribution of sources using BaA/(BaA + Chry) and InP/(InP + BghiP) that petroleum (< 0.20), petroleum combustion (0.20–0.50), and fossil fuel combustion (> 0.35)^18^. In this study, the BaA/(BaA + Chry) fluctuated between 0.04 and 0.97 with an average value of 0.16 (Table 2), which indicates petrogenic sources in all the studied locations except in IIb and IIc, which indicates fossil fuel combustion sources such as proposed wood, coal and grass combustion^22,57^. Unlike seawater samples, the surface sediment of the Nile Delta was occupied by both LMW and HMW rings. The LMW ranged between 8.1 and 76.6% of the ΣPAHs concentration, whereas HMW rings ranged between 23.4 and 91.8% (Table 3). Among the 3 rings of PAHs, Anthracene (Ant) were the dominant member compared to others. Both pyrolytic and petrogenic sources are indicated by the LMW/HMW ratio, which ranges between 0.09 and 3.3. Transport, storage, and use of crude oil and crude oil derivatives have all been frequent sources of petrogenic PAHs entering the environment. Petrogenic PAHs are released into the maritime environment by motor oil and petrol leaks, storage tank breaches, oil spills and tiny petrol releases^58^. Similar to how burning and pyrolysis of coal, oil, gas, trash, wood, and other organic materials are the primary causes of environmental PAH emissions^59^. Same as in seawater samples, BaP represents the highest concentrations among all the studied HMW PAHs compounds.

It is possible to detect the source of PAH fractions by comparing the ratio of low to high molecular weight PAH^7,18^. In the sediment, the ΣTEQ is ranged between 0.002 and 6.84 ng/g (Table 3). To assess the extent of PAH pollution in aquatic environments, the sediment quality guidelines (SQGs)^57^ were widely applied. Sediment quality may be determined using the effective range low (ERL) and effective range medium (ERM), according to^60^. The ERL and ERM of these two sediment quality standards denote the low range of adverse biological effects and the above-mentioned or often occurring adverse biological effects, respectively^35^.

The overall values obtained, according to SQGs, were lower than the ERL and ERM values in Table S2 (Supplementary material). The highest ΣPAH_CARC_ value was recorded at IIa station with a value of 13.77 ng/g, while the highest carcinogenic percentage (% ΣPAH_CARC_ = 88.27%) was reported at Vc station (Table 3).

Unlike seawater, the ratio of % ΣCOMB/PAHs in the sediment samples differ from % ΣCARC/PAHs. The greatest carcinogenic PAH percentage (% ΣPAH_COMB_ = 91.95%) was found at VIIa, whereas the highest COMB concentration value (concentration of 14.59 ng/g) was found at IIa station. An same pyrolytic source to that found in the seawater samples was found in this study's sediment, where the Ant/(Ant + Phe) ratio is higher than 0.1 (Table 3). On the other hands, the ratio of BaA/(BaA + Chr) was ranged between 0.001 and 0.92 with an average value of 0.09, which indicate petrogenic sources in all stations except in station Ib and IVa, which is > 0.35 and that indicates that the PAH in the two studied sites were mainly resulting from combustion sources^61^.

Using cluster analysis, it was possible to assess how the distribution patterns of the PAHs and *n*-alkanes from the sample locations were comparable. According to Hori et al.^16^, the analysis's algorithm was based on Ward's technique. The obtained dendrogram is shown in Fig. S1 (supplementary data). For seawater and sediment samples, Sites Ia–VIIIc were divided into 2 clusters (IIIa–IIIb). While site IIIb had sites with comparatively high *n*-alkane compositions, site IIIa had five locations with relatively low compositions for seawater. Six sites in site IIIa had relatively low PAH contents, whereas sites in site IIIb had quite high PAH concentrations. Contrary to the seawater samples, site IIIa silt comprised eight locations with *n*-alkane compositions that were relatively high, as well as eight sites with PAH concentrations that were reasonably high. The correlation matrix between *n*-alkane and PAHs in seawater and between *n*-alkanes, PAHs and PSA (fractions %) are present in Tables S2 and S3. In seawater samples, a significant correlation (*r* = 0.468–0.909, p < 0.05) was among the low molecular weight *n*-alkanes, such as C9, C10, C11,C12, C13, C15, C18, C20 as well as PAHs between Ant and Phe. In sediment, a significant correlation (r = 0.593–0.999, p < 0.05) was among *n*-alkanes, such as C10, C13, C14, C15, C18, C20, as well as PAHs between Acy, Flu, Phe, Ant and Chry (r = 0.776–0.949, p < 0.05). According to their strong correlation values, their molecules could originate from the same emission sources (Tables S9 and S10 in supplementary data).

The percentages of sand and clay at the two study sites^26^ generally revealed significant variances in the wet PSA analysis's findings. The sand percentage varied from 3.56% at station VIIc to 95.49% at station Va. Conversely, the clay percentage varied from 0.27% at station Vc to 12.50% at station IVc. Between 3.95 and 95% of the coastal near-shore stations (IIa, IIb, IIIb, IVa, Va, VIIa, and VIIb), which were located between 10 and 30 m of depth, were made up of fine silt and sand (Table S11 in supplementary data).

The Σ*n*-alkanes and ΣPAHs concentration levels in the surficial sediments and seawaters in the the studied areas compared with other coastal studies in the Mediterranean Sea and other world regions are present in Table 4.Generally, the ΣPAHs concentrations in the sediments of the studied areas were extremely lower (1.23 to 27.55 ngg^−1^) than El-Mex Bay Alexandria coast, Egypt, (1478–1637 ng g^−1^)^64^ and Abu Qir Bay Alexandria coast, Egypt (69–1464 ng g^−1^)^65^ as shown in Table 4. on the other hand the Σ*n*-alkanes concentrations in the sediments of the studied areas were higher that studied by^75,76^ near oil filed area in Iraq.

**Table 4:** The Σn-alkanes and ΣPAHs concentration levels in surficial sediments and seawaters in the Nile Delta coast of the Mediterranean Sea compared with other coastal studies in the Mediterranean Sea and other world region.

Study area	Compounds	Average (Range)	Reference
Sediment (ng/g dw for PAHs and µg/g dw for *n*-Alkanes)			
Nile Delta coast of the Mediterranean Sea, Egypt	Σ*n*-Alkanes	31.02 ± 27.96 (4.71–84.28)	This study
Nile Delta coast of the Mediterranean Sea, Egypt	Σ13PAHs	8.32 ± 7.36 (1.23–27.55)	This study
Nile Delta, Egypt	Σ13PAHs	27.89 ± 49.82 (4.55–207.48)	^62^
Egyptian Mediterranean Coast	Σ16PAHs	25,046 (13,156–34,852)	^63^
El-Mex Bay Alexandria coast, Egypt	Σ16PAHs	(1478—1637)	^64^
Abu Qir Bay Alexandria coast, Egypt	Σ16PAHs	(69–1464)	^65^
Egyptian Coast Coastline	Σ16PAHs	(3.5–14,100)	^66^
Egyptian Coastline from	Σ16PAHs	530 (208–1020)	^67^
Mediterranean Coast Napoli (Italy)	Σ16PAHs	(435–872)	^68^
Tunisia, Southern Mediterranean Sea	Σ16PAHs	(175–10,769)	^69^
Milazzo Gulf (Italy) Coastal line	Σ19PAHs	492 (5.6–7402)	^70^
Eastern basin Mediterranean Sea	Σ24PAHs	(2.2–1056.2)	^71^
French and Spanish coasts	Σ18PAHs	(˃1–8500)	^72^
Jiaozhou Bay, China	–	(37.7–290.9)	^73^
Ulsan Bay, Korea	–	(35 -1300)	^74^
Nile Delta, Egypt	Σ*n*-Alkanes	51.98 ± 17.49 (18.85 to 164.37)	^62^
Shatt Al-Arab River, Iraq	Σ*n*-Alkanes	4.76 – 10.09	^75^
Shatt Al-Arab River, Iraq	Σ*n*-Alkanes	0.244–8.243	^76^
West Qurna-2 Oil field, Iraq	Σ*n*-Alkanes	4.999–43.324	^77^
Al-Hammar Marsh, Iraq	Σ*n*-Alkanes	6.176–8.835	^78^
Water (µg/L) (µg/L for PAHs and mg/L for *n*-Alkanes)			
Liaodong Bay	Σ*n*-Alkanes	145.96–896.58	^79^
Jiaozhou Bay	Σ*n*-Alkanes	23.6–86.2	^80^
Kongsfjorden	Σn-Alkanes	33.4–79.8	^81^
Daya Bay	Σn-Alkanes	4228–29,325	^82^

## Conclusion

This study gives baseline data around the distribution and sources of *n*-alkanes and PAHs residue in the seawater and sediment samples collected from the Nile delta coasts of the Mediterranean. The overall mean value of *n*-alkanes concentrations has been reported to be higher in seawater samples than in sediment samples, proving the presence of new inputs of *n*-alkanes at the studied sites. The total average concentration of 13 PAHs in seawater was very close to the concentration reported in the sediment samples. The CPI values show that the terrestrial plant waxes are the main source of *n*-alkanes of three locations (IIIb, Va, and VIc). Where at the rest of the stations were CPI < 1, which indicates aquatic material sources such as bacteria and algae for *n*-alkanes in the sediment samples. The greatest high CPI value is found in station VIb with CPI > 2, which may also reflect the contribution of the natural sources. While CPI values close to 1 in IVc and VIc sites are accompanied with automobile emissions and other activities. In seawater, Ace was the dominant member compared to others LMW PAHs in seawater samples, and Ant were the dominant member compared to others LMW PAHs in the sediment. The LMW/HMW ratio is less than 1 in all the studied seawater samples, which indicates pyrolytic sources. In the seawater samples, the ΣTEQ ranged between 3.7 and 13.5 µg/L, while in the sediment samples, the ΣTEQ ranged between 0.002 and 6.84 ng/g. In seawater, the highest carcinogenic PAH percentage was 88.66%, while in the sediment the highest carcinogenic PAH percentage was 88.27%. The Ant/(Ant + Phe) ratio in sediment indicated a pyrolytic source same as in seawater samples. On the other hands, the BaA/(BaA + Chry) ratio ranged between 0.001 and 0.918 with an average of 0.09, which indicates petrogenic sources in all stations except in station Ib and IVa, which is > 0.35 and that mainly derived from combustion sources.

### Supplementary Information

Supplementary Information.

## Data Availability

The corresponding author of the study can provide access to the datasets utilized in this inquiry upon request.
