# Supplementary material for: Sources of hydrocarbons and their risk assessment in seawater and sediment samples collected from the Nile Delta coast of the Mediterranean Sea
Source: Sci Rep. 2024 Mar 1;14:5082. doi: 10.1038/s41598-024-55339-4 (PMC10907701; doi:10.1038/s41598-024-55339-4)
Supplement: Supplementary file 1 — Supplementary Information. [file 41598_2024_55339_MOESM1_ESM.docx]

**Supplementary data**

**Sources of hydrocarbons and their risk assessment in seawater and sediment samples collected from the Nile Delta coast of the Mediterranean Sea**

Mohamed A. Hassaan, Safaa Ragab, Amany El Sikaily, Ahmed El Nemr*

Table S1. The sample stations are described below (Depth and geographic location).

| No. | Location | Long | Lat | Depth | Area | | |
| --- | --- | --- | --- | --- | --- | --- | --- |
| 1 | Ia | 29.8846052 | 31.22092062 | 10 | Western Harbor | | |
| 2 | Ib | 29.88072909 | 31.24805334 | 30 |  |  |  |
| 3 | Ic | 29.77995043 | 31.29069046 | 50 |  |  |  |
| 4 | IIa | 30.12879966 | 31.39922133 | 10 | Abu Qir | | |
| 5 | IIb | 30.07453422 | 31.44961067 | 30 |  |  |  |
| 6 | IIc | 30.00476438 | 31.54263713 | 50 |  |  |  |
| 7 | IIIa | 30.26446325 | 31.59302646 | 10 | Rasheed Nile branch | | |
| 8 | IIIb | 30.31097648 | 31.67830072 | 30 |  |  |  |
| 9 | IIIc | 30.29934817 | 31.75582277 | 50 |  |  |  |
| 10 | IVa | 30.65594960 | 31.59302646 | 10 | Burullus | | |
| 11 | IVb | 30.60168417 | 31.71318564 | 30 |  |  |  |
| 12 | IVc | 30.55517094 | 31.83334482 | 50 |  |  |  |
| 13 | Va | 30.99704663 | 31.64729190 | 10 | Balteem | | |
| 14 | Vb | 30.99317052 | 31.73644225 | 30 |  |  |  |
| 15 | Vc | 30.98929442 | 31.84497313 | 50 |  |  |  |
| 16 | VIa | 31.43117011 | 31.54651323 | 10 | Mansourah elgedida and Gamasa | | |
| 17 | VIb | 31.45055062 | 31.68605292 | 30 |  |  |  |
| 18 | VIc | 31.45442672 | 31.81396431 | 50 |  |  |  |
| 19 | VIIa | 31.84203698 | 31.56201764 | 10 | Dameitta Nile branch Ras elbar | | |
| 20 | VIIb | 31.86141749 | 31.67830072 | 30 |  |  |  |
| 21 | VIIc | 31.87304580 | 31.75194666 | 50 |  |  |  |
| 22 | VIIIa | 32.10948805 | 31.48837169 | 10 | Manzala Lake | | |
| 23 | VIIIb | 32.17538180 | 31.61628308 | 30 |  |  |  |
| 24 | VIIIc | 32.22964723 | 31.72093784 | 50 |  |  |  |

Table S2. The abbreviation, molecular formula, molecular weight (MW) and carcinogenicity index of PAHs [1,2].

| Compound name (IUPAC) | No. of rings | Abb. | Molecular formula | MW | ERL | ERM | Car.[3] | CP [4] | TEF, [5] |
| --- | --- | --- | --- | --- | --- | --- | --- | --- | --- |
| Acenaphthylene | 3 | Acy | C_12_H_8_ | 152.2 | 44 | 640 | 0 | _ |  |
| Phenanthrene | 3 | Phe | C_14_H_10_ | 178.2 | 240 | 1500 | 0 | D |  |
| Anthracene | 3 | Ant | C_14_H_10_ | 178.2 | 853 | 1100 | 0 | D |  |
| Fluoranthene | 4 | Flu | C_16_H_10_ | 202.2 | 600 | 5100 | 0 | D |  |
| Pyrene | 4 | Pyr | C_16_H_10_ | 202.2 | 665 | 2600 | 0 | D |  |
| Benzo(a)anthracene | 4 | BaA | C_18_H_12_ | 228.3 | 261 | 1600 | ++ | B2 | 0.1 |
| Chrysene | 4 | Chry | C_18_H_12_ | 228.3 | 384 | 2800 | ++ | B2 | 0.001 |
| Benzo(b)fluoranthene | 5 | BbF | C_20_H_12_ | 252.3 | 320 | 1800 | ++ | B2 | 0.1 |
| Benzo(k)fluoranthene | 5 | BkF | C_20_H_12_ | 252.3 | 280 | 1620 | ++ | B2 | 0.01 |
| Benzo(a)Pyrene | 5 | BaP | C_20_H_12_ | 252.3 | 430 | 1600 | +++ | B2 | 1 |
| Dibenzo(a,h)anthracene | 5 | DahA | C_22_H_14_ | 278.3 | 63.4 | 260 | +++ | B2 | 1 |
| Benzo(g,h,i)perylene | 6 | BghiP | C_22_H_12_ | 276.3 | 430 | 1600 | 0.000 | D |  |
| Indeno(1,2,3-cd)pyrene | 6 | InP | C_22_H_12_ | 276.3 | _ | _ | +++ | B2 | 0.1 |

Table S3. The SRM and electron ionization energies used for 13 PAHs.

| PAH | Analytes ID | RT (min) | Precursor ion mass | Product ion mass | Collision Energy (eV) | Limit of detection (pg) |
| --- | --- | --- | --- | --- | --- | --- |
| Acenaphthylene | Ace | 8.98 | 152.10 | 151.00 | 20 | 2.5 |
| Fluorene | Flu | 11.07 | 166.00 | 165.00 | 30 | 2.5 |
| Phenanthrene | Phe | 14.60 | 178.00 | 152.00 | 25 | 1.0 |
| Anthracene | Ant | 14.60 | 178.00 | 152.00 | 20 | 1.0 |
| Pyrene | Pyr | 20.49 | 202.10 | 201.00 | 20 | 1.0 |
| Benz(a)anthracene | BaA | 26.15 | 228.10 | 226.00 | 35 | 2.5 |
| Chrysene | Chry | 26.15 | 228.10 | 226.00 | 35 | 1.0 |
| Benzo(b)fluoranthene | BbF | 31.00 | 252.10 | 250.00 | 35 | 1.0 |
| Benzo(k)fluoranthene | BkF | 31.00 | 252.10 | 250.00 | 35 | 1.0 |
| Benzo(a)pyrene | BaP | 31.00 | 252.10 | 250.00 | 35 | 1.0 |
| Dibenzo(a.h)anthracene | DahA | 36.30 | 278.10 | 274.10 | 60 | 5.0 |
| Indeno(1.2.3.cd)pyrene | InP | 36.40 | 276.10 | 272.00 | 60 | 5.0 |
| Benzo(g.h.i)perylene | BghiP | 36.40 | 276.10 | 272.00 | 60 | 2.5 |

Table S4. The SRM and electron ionization energies used for *n*-alkanes C9 to C20.

| *n*-alkaine | RT (min) | Precursor ion mass | Product ion mass | Collision Energy (eV) | Limit of detection (ng) |
| --- | --- | --- | --- | --- | --- |
| C-9 | 3.88 | 128.17 | 57.12 | 10 | 2.5 |
| C-10 | 5.07 | 142.18 | 57.12 | 10 | 2.5 |
| C-11 | 6.25 | 156.20 | 57.12 | 10 | 1.0 |
| C-12 | 7.32 | 170.23 | 57.12 | 10 | 1.0 |
| C-13 | 8.32 | 184.23 | 57.12 | 15 | 1.0 |
| C-14 | 9.24 | 198.25 | 57.11 | 15 | 1.0 |
| C-15 | 10.12 | 212.23 | 57.12 | 15 | 1.0 |
| C-16 | 10.94 | 226.29 | 57.11 | 15 | 2.0 |
| C-17 | 11.71 | 240.31 | 57.13 | 15 | 2.0 |
| C-18 | 12.44 | 254.34 | 57.12 | 15 | 2.0 |
| C-19 | 13.14 | 268.34 | 57.12 | 15 | 3.0 |
| C-20 | 13.81 | 282.32 | 57.12 | 15 | 3.0 |

Table S5. Average concentrations of *n*-alkanes in water (mg/L)

| Compounds  Site | C-9 | C-10 | C-11 | C-12 | C-13 | C-14 | C-15 | C-16 | C-17 | C-18 | C-19 | C-20 |
| --- | --- | --- | --- | --- | --- | --- | --- | --- | --- | --- | --- | --- |
| Ia | 2.56 | 3.19 | 4.17 | 3.36 | 1.42 | 4.61 | 1.60 | 5.26 | 5.06 | 4.58 | 3.51 | 4.91 |
| Ib | 2.11 | 3.58 | 4.74 | 2.90 | 1.42 | 4.54 | 1.54 | 5.02 | 4.54 | 4.81 | 2.80 | 2.84 |
| Ic | 2.72 | 3.25 | 5.20 | 4.75 | 2.16 | 5.12 | 2.25 | 5.77 | 4.72 | 5.64 | 2.89 | 4.48 |
| IIa | 3.37 | 3.51 | 5.63 | 6.81 | 3.09 | 5.37 | 2.31 | 6.25 | 4.68 | 5.60 | 3.62 | 3.72 |
| IIb | 2.41 | 2.72 | 4.68 | 4.62 | 2.00 | 4.77 | 2.13 | 5.91 | 3.42 | 6.35 | 3.21 | 4.42 |
| IIc | 2.59 | 3.84 | 5.50 | 8.59 | 2.44 | 5.09 | 2.84 | 6.51 | 16.59 | 7.82 | 6.11 | 4.79 |
| IIIa | 4.01 | 3.25 | 4.98 | 5.75 | 2.39 | 4.94 | 2.27 | 5.24 | 5.31 | 4.98 | 2.75 | 3.23 |
| IIIb | 3.34 | 3.75 | 5.16 | 5.56 | 2.09 | 4.86 | 3.46 | 5.22 | 12.12 | 5.52 | 5.85 | 4.00 |
| IIIc | 3.63 | 3.57 | 4.60 | 5.13 | 2.00 | 4.66 | 1.74 | 4.92 | 4.25 | 3.77 | 2.56 | 2.03 |
| IVa | 4.05 | 4.16 | 5.85 | 7.83 | 2.50 | 5.03 | 2.88 | 5.35 | 7.85 | 4.23 | 2.35 | 2.48 |
| IVb | 3.10 | 3.06 | 4.35 | 4.58 | 1.70 | 4.51 | 1.43 | 4.54 | 2.42 | 1.94 | 1.64 | 1.34 |
| IVc | 1.54 | N/A | 3.14 | N/A | 0.09 | 3.73 | 0.17 | 3.38 | N/A | N/A | N/A | N/A |
| Va | 2.47 | 2.79 | 4.43 | 0.92 | 4.24 | 1.69 | 4.77 | 3.92 | N/A | N/A | 0.09 | N/A |
| Vb | 3.44 | 2.88 | 3.49 | 2.47 | 0.82 | 4.33 | 1.81 | 4.91 | 4.92 | 4.45 | 4.86 | 4.19 |
| Vc | 4.98 | 3.78 | 4.35 | 7.37 | 1.33 | 4.83 | 3.49 | 6.29 | 12.67 | 9.84 | 9.54 | 8.58 |
| VIa | 3.21 | 2.70 | 3.51 | 2.26 | 0.61 | 3.71 | 0.95 | 4.31 | 2.82 | 3.58 | 3.10 | 2.54 |
| VIb | 4.30 | 3.01 | 3.87 | 3.38 | 1.02 | 4.41 | 2.13 | 4.95 | 3.57 | 6.02 | 5.65 | 3.56 |
| VIc | 4.10 | 4.22 | 4.97 | 3.13 | 1.02 | 4.46 | 5.00 | 5.93 | 19.45 | 7.43 | 5.34 | 7.12 |
| VIIa | 5.07 | 3.39 | 4.71 | 4.59 | 0.14 | 4.73 | 2.13 | 5.51 | 6.82 | 6.46 | 6.03 | 6.79 |
| VIIb | 3.53 | 2.81 | 3.72 | 2.01 | 0.68 | 4.10 | 2.01 | 4.57 | 3.11 | 4.47 | 3.81 | 2.90 |
| VIIc | 4.17 | 3.30 | 3.87 | 4.08 | 0.81 | 4.37 | 1.97 | 4.69 | 6.68 | 6.34 | 3.99 | 3.07 |
| VIIIa | 5.56 | 4.09 | 4.53 | 7.02 | 1.81 | 5.12 | 5.12 | 7.45 | 18.53 | 13.21 | 10.29 | 10.78 |
| VIIIb | 3.92 | 3.00 | 3.87 | 3.74 | 0.59 | 4.44 | 1.94 | 5.41 | 4.75 | 7.58 | 7.58 | 6.40 |
| VIIIc | 5.24 | 3.38 | 4.11 | 5.89 | 1.12 | 4.72 | 2.69 | 5.99 | 6.58 | 8.55 | 9.69 | 7.00 |
| MIN | 1.54 | 2.70 | 3.14 | 0.92 | 0.09 | 1.69 | 0.17 | 3.38 | 2.42 | 1.94 | 0.09 | 1.34 |
| MAX | 5.56 | 4.22 | 5.85 | 8.59 | 4.24 | 5.37 | 5.12 | 7.45 | 19.45 | 13.21 | 10.29 | 10.78 |
| Average | 3.56 | 3.36 | 4.48 | 4.64 | 1.56 | 4.51 | 2.44 | 5.30 | 7.31 | 6.05 | 4.66 | 4.60 |
| SD | 1.04 | 0.46 | 0.71 | 2.00 | 0.97 | 0.73 | 1.21 | 0.89 | 5.14 | 2.41 | 2.65 | 2.32 |

Table S6. Average concentrations of PAHs and ΣPAHs in water (µg/L).

| Compounds/  Stations | Pry | Ace | Flu | DabA | Ant | Phe | Chry | BghiP | BaA | BbF | BkF | BaP | I nP | ΣPAHs |
| --- | --- | --- | --- | --- | --- | --- | --- | --- | --- | --- | --- | --- | --- | --- |
| Ia | N/A | 2.19 | 0.33 | N/A | 0.00 | 0.01 | 0.08 | N/A | 0.00 | 0.01 | 0.01 | 12.87 | N/A | 15.49 |
| Ib | N/A | 2.69 | 0.01 | N/A | 0.00 | 0.00 | 0.03 | N/A | 0.00 | 0.00 | 0.01 | 6.12 | N/A | 8.86 |
| Ic | N/A | 0.46 | 0.41 | N/A | 0.49 | 0.08 | 0.05 | N/A | 0.00 | 0.00 | 0.03 | 3.77 | N/A | 5.30 |
| IIa | N/A | 2.40 | 0.29 | N/A | 0.22 | 0.01 | 0.05 | N/A | 0.00 | 0.00 | 0.02 | 11.01 | N/A | 14.01 |
| IIb | N/A | 0.50 | 0.28 | N/A | 0.33 | 0.04 | 0.07 | N/A | 2.10 | 0.00 | 0.01 | 6.62 | N/A | 9.94 |
| IIc | N/A | 2.41 | 0.30 | N/A | 0.23 | 0.04 | 0.03 | N/A | 0.28 | 0.01 | 0.03 | 6.38 | N/A | 9.70 |
| IIIa | N/A | 2.54 | 0.59 | N/A | 0.79 | 0.03 | 0.07 | N/A | 0.00 | 0.01 | 0.02 | 12.29 | N/A | 16.34 |
| IIIb | N/A | 2.39 | 0.66 | N/A | 1.00 | 0.03 | 0.07 | N/A | 0.00 | 0.00 | 0.01 | 6.46 | N/A | 10.61 |
| IIIc | N/A | 0.58 | 0.23 | N/A | 0.06 | 0.01 | 0.02 | N/A | 0.00 | 0.00 | 0.01 | 4.12 | N/A | 5.02 |
| IVa | N/A | 1.39 | 0.62 | N/A | 0.35 | 0.07 | 0.03 | N/A | 0.00 | 0.00 | 0.01 | 7.19 | N/A | 9.66 |
| IVb | N/A | 0.48 | 0.16 | N/A | 0.01 | 0.01 | 0.08 | N/A | 0.01 | 0.00 | 0.02 | 3.72 | N/A | 4.49 |
| IVc | N/A | 1.56 | 0.20 | N/A | 0.00 | 0.01 | 0.03 | N/A | 0.00 | 0.00 | 0.01 | 4.93 | N/A | 6.75 |
| Va | N/A | 1.67 | 1.39 | N/A | 0.00 | 0.01 | 0.02 | N/A | 0.00 | 0.00 | 0.01 | 9.92 | N/A | 13.02 |
| Vb | N/A | 1.44 | 1.80 | N/A | 0.01 | 0.01 | 0.02 | N/A | 0.00 | 0.01 | 0.01 | 6.24 | N/A | 9.53 |
| Vc | N/A | 1.59 | 1.23 | N/A | 0.00 | 0.02 | 0.04 | N/A | 0.00 | 0.00 | 0.01 | 4.26 | N/A | 7.15 |
| VIa | N/A | 1.59 | 0.81 | N/A | 0.00 | 0.01 | 0.02 | N/A | 0.01 | 0.00 | 0.01 | 13.48 | N/A | 15.93 |
| VIb | N/A | 1.34 | 1.35 | N/A | 0.01 | 0.01 | 0.02 | N/A | 0.00 | 0.00 | 0.01 | 3.80 | N/A | 6.52 |
| VIc | N/A | 0.62 | 1.47 | N/A | 0.00 | 0.01 | 0.04 | N/A | 0.00 | 0.00 | 0.01 | 4.07 | N/A | 6.22 |
| VIIa | N/A | 0.49 | 1.38 | N/A | 0.01 | 0.01 | 0.02 | N/A | 0.00 | 0.01 | 0.02 | 4.00 | N/A | 5.94 |
| VIIb | N/A | 0.74 | 0.72 | N/A | 0.01 | 0.01 | 0.03 | N/A | 0.00 | 0.00 | 0.01 | 3.79 | N/A | 5.30 |
| VIIc | N/A | 0.24 | 1.46 | N/A | 0.01 | 0.01 | 0.05 | N/A | 0.00 | 0.00 | 0.03 | 5.80 | N/A | 7.59 |
| VIIIa | N/A | 1.25 | 2.17 | N/A | 0.34 | 0.01 | 0.05 | N/A | 0.00 | 0.00 | 0.01 | 4.77 | N/A | 8.60 |
| VIIIb | N/A | 0.28 | 1.58 | N/A | 0.01 | 0.00 | 0.04 | N/A | 0.00 | 0.00 | 0.01 | 12.24 | N/A | 14.16 |
| VIIIc | N/A | 0.53 | 1.80 | N/A | 0.01 | 0.01 | 0.04 | N/A | 0.00 | 0.00 | 0.01 | 8.80 | N/A | 11.20 |
| MIN | N/A | 0.24 | 0.01 | N/A | 0.00 | 0.00 | 0.02 | N/A | 0.00 | 0.00 | 0.01 | 3.72 | N/A | 4.49 |
| MAX | N/A | 2.69 | 2.17 | N/A | 1.00 | 0.08 | 0.08 | N/A | 2.10 | 0.01 | 0.03 | 13.48 | N/A | 16.34 |
| Average | N/A | 1.31 | 0.88 | N/A | 0.16 | 0.02 | 0.04 | N/A | 0.10 | 0.01 | 0.01 | 6.94 | N/A | 9.47 |
| SD | N/A | 0.81 | 0.64 | N/A | 0.27 | 0.02 | 0.02 | N/A | 0.43 | 0.00 | 0.01 | 3.28 | N/A | 3.69 |

Table S7. Average concentrations of *n*-Alkanes in Sediment (µg/g)

| Compounds  Stations | C-9 | C-10 | C-11 | C-12 | C-13 | C-14 | C-15 | C-16 | C-17 | C-18 | C-19 | C-20 |
| --- | --- | --- | --- | --- | --- | --- | --- | --- | --- | --- | --- | --- |
| Ib | 0.38 | 0.56 | 0.79 | 0.37 | 0.45 | 0.41 | 0.33 | 0.54 | 1.08 | 1.02 | 0.97 | 0.75 |
| Ic | 0.37 | 2.22 | 3.32 | 2.40 | 2.40 | 4.90 | 3.03 | 5.12 | 2.08 | 11.76 | 1.70 | 12.77 |
| IIa | 0.66 | 3.99 | 1.28 | 5.87 | 4.60 | 7.73 | 5.25 | 9.50 | 3.44 | 17.94 | 2.92 | 20.87 |
| IIb | 0.50 | 2.74 | 0.94 | 4.39 | 3.56 | 5.93 | 4.06 | 7.76 | 2.40 | 14.85 | 2.05 | 16.77 |
| IIc | 0.47 | 3.02 | 1.05 | 4.96 | 3.99 | 6.81 | 4.78 | 8.79 | 2.81 | 16.81 | 2.29 | 19.06 |
| IIIa | 0.18 | 1.08 | 0.56 | 2.79 | 2.44 | 4.75 | 3.03 | 6.49 | 1.73 | 12.48 | 1.81 | 14.15 |
| IIIb | 0.45 | 2.58 | 0.89 | 3.82 | 3.00 | 5.21 | 3.47 | 6.84 | 2.06 | 12.68 | 1.95 | 14.65 |
| IIIc | 0.39 | 2.27 | 0.78 | 3.16 | 2.37 | 4.12 | 2.64 | 4.24 | 2.64 | 9.75 | 1.39 | 11.17 |
| IVa | 0.49 | 0.67 | 0.91 | 0.43 | 0.42 | 0.42 | 0.23 | 0.40 | 0.67 | 0.53 | 0.54 | 0.47 |
| IVb | 0.20 | 0.71 | 0.51 | 2.02 | 1.96 | 4.17 | 2.79 | 5.86 | 1.79 | 11.17 | 1.68 | 11.95 |
| IVc | 0.51 | 0.74 | 1.05 | 0.68 | 0.60 | 0.58 | 0.33 | 0.54 | 0.85 | 0.77 | 0.75 | 0.73 |
| Va | 0.41 | 0.52 | 0.69 | 0.25 | 0.30 | 0.36 | 0.16 | 0.35 | 0.40 | 0.40 | 0.47 | 0.41 |
| Vb | 0.49 | 2.79 | 0.94 | 3.88 | 2.84 | 5.04 | 3.36 | 6.55 | 3.45 | 12.07 | 2.04 | 12.94 |
| Vc | 0.33 | 0.57 | 0.77 | 0.59 | 0.62 | 0.61 | 0.42 | 0.58 | 1.15 | 0.76 | 0.64 | 0.59 |
| VIa | 0.54 | 0.68 | 1.01 | 0.55 | 0.55 | 0.48 | 0.35 | 0.47 | 0.59 | 0.61 | 0.62 | 0.68 |
| VIb | 0.80 | 2.48 | 1.80 | 0.68 | 0.56 | 0.50 | 1.18 | 0.81 | 9.87 | 1.56 | 1.35 | 1.13 |
| VIc | 0.41 | 0.60 | 0.89 | 0.52 | 0.54 | 0.25 | 0.26 | 0.48 | 0.38 | 0.61 | 0.44 | 0.49 |
| VIIa | 0.47 | 0.67 | 0.77 | 0.34 | 0.40 | 0.39 | 0.30 | 0.41 | 0.61 | 0.46 | 0.41 | 0.43 |
| VIIb | 0.56 | 0.75 | 0.94 | 0.46 | 0.46 | 0.42 | 0.28 | 0.40 | 0.53 | 0.44 | 0.34 | 0.36 |
| VIIc | 0.45 | 0.63 | 0.93 | 0.46 | 0.44 | 0.42 | 0.24 | 0.40 | 0.55 | 0.46 | 0.45 | 0.35 |
| VIIIa | 0.38 | 1.60 | 1.44 | 0.56 | 0.47 | 0.58 | 0.91 | 0.83 | 4.23 | 1.73 | 1.30 | 1.26 |
| VIIIb | 0.37 | 0.59 | 0.77 | 0.46 | 0.44 | 0.52 | 0.32 | 0.53 | 0.48 | 0.74 | 0.48 | 0.58 |
| VIIIc | 0.59 | 3.53 | 1.12 | 5.37 | 4.16 | 7.01 | 4.61 | 6.81 | 2.84 | 16.30 | 2.41 | 17.77 |
| MIN | 0.18 | 0.52 | 0.51 | 0.25 | 0.30 | 0.25 | 0.16 | 0.35 | 0.38 | 0.40 | 0.34 | 0.35 |
| MAX | 0.80 | 3.99 | 3.32 | 5.87 | 4.60 | 7.73 | 5.25 | 9.50 | 9.87 | 17.94 | 2.92 | 20.87 |
| Average | 0.45 | 1.56 | 1.05 | 1.96 | 1.63 | 2.68 | 1.84 | 3.25 | 2.03 | 6.34 | 1.26 | 6.97 |
| SD | 0.14 | 1.13 | 0.57 | 1.90 | 1.46 | 2.71 | 1.78 | 3.34 | 2.06 | 6.73 | 0.78 | 7.68 |

Table S8. Average concentrations of PAHs in the Sediment samples (ng/g).

| Compounds/  Station | Pyr | Da,hA | InP | BghiP | Ace | Flu | Ant | Phe | Chry | BaA | BbF | BkF | BaP | ΣPAHs |
| --- | --- | --- | --- | --- | --- | --- | --- | --- | --- | --- | --- | --- | --- | --- |
| Ib | 0.08 | N/A | N/A | N/A | 0.09 | 0.06 | 0.10 | 0.09 | N/A | 0.02 | N/A | N/A | 0.89 | 1.32 |
| Ic | 0.38 | N/A | N/A | N/A | 1.15 | 1.07 | 2.27 | 1.57 | 2.13 | 0.00 | N/A | N/A | N/A | 8.58 |
| IIa | 0.83 | N/A | N/A | N/A | 3.47 | 1.69 | 4.83 | 3.79 | 7.34 | 0.01 | N/A | N/A | 6.42 | 28.38 |
| IIb | 0.55 | N/A | N/A | N/A | 2.92 | 1.53 | 3.17 | 2.30 | 4.36 | 0.01 | N/A | N/A | 2.84 | 17.68 |
| IIc | 0.62 | N/A | N/A | N/A | 3.32 | 1.68 | 4.14 | 3.02 | 4.45 | N/A | N/A | N/A | 6.83 | 24.07 |
| IIIa | 0.43 | N/A | N/A | N/A | 2.45 | 1.38 | 2.76 | 1.71 | 3.03 | N/A | N/A | N/A | 3.19 | 14.96 |
| IIIb | 0.58 | N/A | N/A | N/A | 1.34 | 1.36 | 2.61 | 1.60 | 4.34 | 0.02 | N/A | N/A | 3.55 | 15.39 |
| IIIc | 0.33 | N/A | N/A | N/A | 1.05 | 0.96 | 1.72 | 0.45 | 2.90 | 0.00 | N/A | N/A | 1.83 | 9.25 |
| IVa | 0.22 | N/A | N/A | N/A | 0.53 | 0.04 | 0.13 | 0.08 | 0.00 | 0.04 | N/A | N/A | 2.14 | 3.18 |
| IVb | 0.39 | N/A | N/A | N/A | 1.84 | 0.96 | 2.18 | 2.14 | 1.79 | 0.02 | N/A | N/A | 2.97 | 12.29 |
| IVc | 0.10 | N/A | N/A | N/A | 0.36 | 0.07 | 0.17 | 0.12 | 2.76 | 0.06 | N/A | N/A | 1.08 | 4.71 |
| Va | 0.15 | N/A | N/A | N/A | 0.30 | 0.04 | 0.07 | 0.05 | 1.78 | 0.03 | N/A | N/A | 1.63 | 4.05 |
| Vb | 0.39 | N/A | N/A | N/A | 1.84 | 1.11 | 2.63 | 2.44 | 1.67 | N/A | N/A | N/A | 0.40 | 10.47 |
| Vc | 0.15 | N/A | N/A | N/A | 0.20 | 0.07 | 0.21 | 0.12 | 3.35 | 0.07 | N/A | N/A | 2.20 | 6.37 |
| VIa | 0.18 | N/A | N/A | N/A | 0.38 | 0.05 | 0.16 | 0.10 | 0.74 | 0.05 | N/A | N/A | 1.23 | 2.87 |
| VIb | 0.17 | N/A | N/A | N/A | 0.34 | 0.05 | 0.12 | 0.07 | 1.52 | 0.04 | N/A | N/A | 1.18 | 3.50 |
| VIc | 0.13 | N/A | N/A | N/A | 0.27 | 0.04 | 0.12 | 0.08 | 1.46 | 0.03 | N/A | N/A | 0.24 | 2.37 |
| VIIa | 0.12 | N/A | N/A | N/A | 0.06 | 0.03 | 0.09 | 0.06 | 1.08 | 0.02 | N/A | N/A | 1.40 | 2.85 |
| VIIb | 0.10 | N/A | N/A | N/A | 0.24 | 0.03 | 0.09 | 0.05 | 1.17 | 0.02 | N/A | N/A | 0.75 | 2.44 |
| VIIc | 0.14 | N/A | N/A | N/A | 0.20 | 0.03 | 0.10 | 0.07 | 1.36 | 0.03 | N/A | N/A | 0.40 | 2.33 |
| VIIIa | 0.07 | N/A | N/A | N/A | 0.30 | 0.05 | 0.24 | 0.08 | 1.39 | 0.03 | N/A | N/A | 1.11 | 3.27 |
| VIIIb | 0.16 | N/A | N/A | N/A | 0.23 | 0.04 | 0.12 | 0.06 | 1.45 | 0.03 | N/A | N/A | 0.38 | 2.47 |
| VIIIc | 0.50 | N/A | N/A | N/A | 1.75 | 1.25 | 3.86 | 3.98 | 3.16 | 0.01 | N/A | N/A | 0.81 | 15.32 |
| MIN | 0.07 | N/A | N/A | N/A | 0.06 | 0.03 | 0.07 | 0.05 | 0.01 | 0.01 | N/A | N/A | 0.24 | 1.32 |
| MAX | 0.83 | N/A | N/A | N/A | 3.47 | 1.69 | 4.83 | 3.98 | 7.34 | 0.07 | N/A | N/A | 6.83 | 28.38 |
| Average | 0.29 | N/A | N/A | N/A | 1.07 | 0.59 | 1.39 | 1.04 | 2.32 | 0.02 | N/A | N/A | 1.98 | 8.61 |
| SD | 0.21 | N/A | N/A | N/A | 1.10 | 0.66 | 1.59 | 1.32 | 1.68 | 0.02 | N/A | N/A | 1.79 | 7.57 |

| 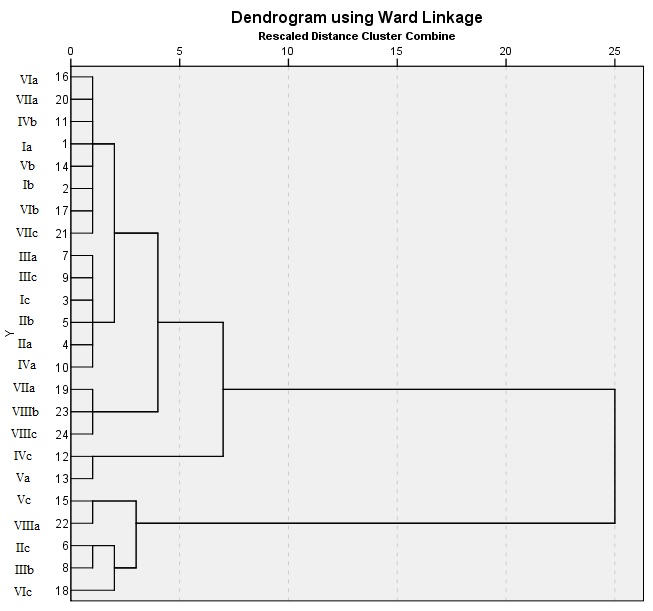 |
| --- |
| (A) |
| 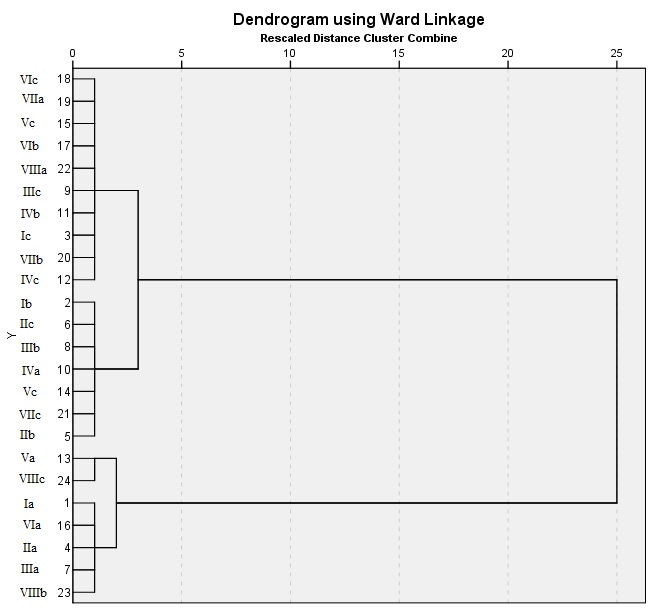 |
| (B) |
| 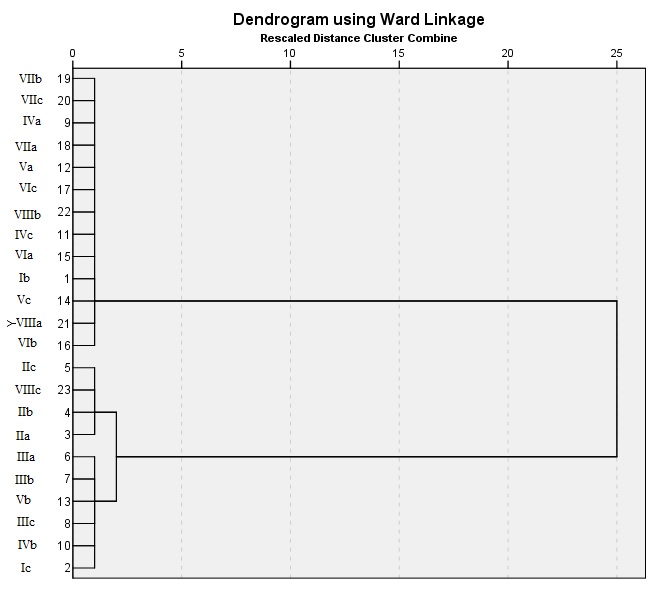 |
| (C) |
| 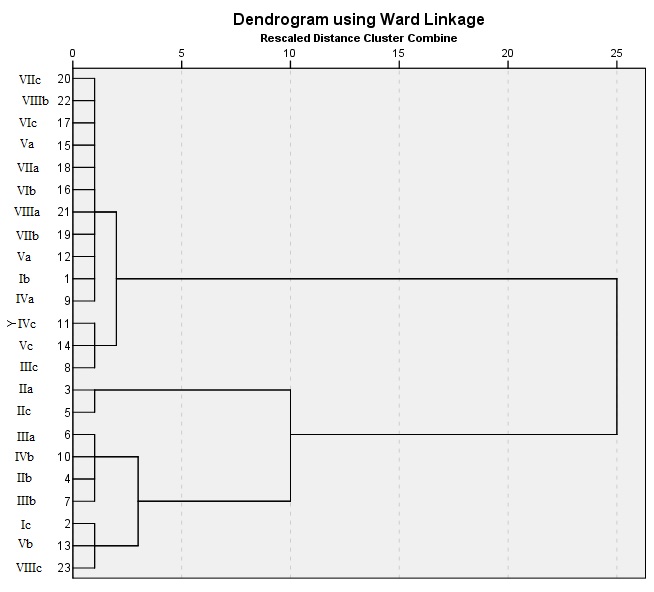 |
| (D) |

Figure S1. Hierarchical cluster analysis dendrograms (Ward’s method) for (A) n-Alkane in water, (B) PAHs in water, (C) n-Alkane in sediment and (D) PAHs in sediment.

Table S9. Correlation matrix between *n*-alkanes, PAHs and PSA at Nile Delta sediment.

|  | C-9 | C-10 | C-11 | C-12 | C-13 | C-14 | C-15 | C-16 | C-17 | C-18 | C-19 | C-20 | Pyr | Ace | Flu | Ant | Phe | Chry | BaA | BbF | BkF | BaP | clay | fine silt | coarse silt | Sand | gravel |
| --- | --- | --- | --- | --- | --- | --- | --- | --- | --- | --- | --- | --- | --- | --- | --- | --- | --- | --- | --- | --- | --- | --- | --- | --- | --- | --- | --- |
| C-9 | 1 |  |  |  |  |  |  |  |  |  |  |  |  |  |  |  |  |  |  |  |  |  |  |  |  |  |  |
| C-10 | .468* | 1 |  |  |  |  |  |  |  |  |  |  |  |  |  |  |  |  |  |  |  |  |  |  |  |  |  |
| C-11 | 0.269 | 0.35 | 1 |  |  |  |  |  |  |  |  |  |  |  |  |  |  |  |  |  |  |  |  |  |  |  |  |
| C-12 | 0.165 | .897** | 0.094 | 1 |  |  |  |  |  |  |  |  |  |  |  |  |  |  |  |  |  |  |  |  |  |  |  |
| C-13 | 0.118 | .878** | 0.133 | .995** | 1 |  |  |  |  |  |  |  |  |  |  |  |  |  |  |  |  |  |  |  |  |  |  |
| C-14 | 0.033 | .847** | 0.159 | .980** | .992** | 1 |  |  |  |  |  |  |  |  |  |  |  |  |  |  |  |  |  |  |  |  |  |
| C-15 | 0.096 | .886** | 0.179 | .980** | .989** | .992** | 1 |  |  |  |  |  |  |  |  |  |  |  |  |  |  |  |  |  |  |  |  |
| C-16 | -.008 | .813** | 0.099 | .961** | .975** | .987** | .985** | 1 |  |  |  |  |  |  |  |  |  |  |  |  |  |  |  |  |  |  |  |
| C-17 | .518* | .593** | 0.385 | 0.281 | 0.26 | 0.249 | 0.352 | 0.258 | 1 |  |  |  |  |  |  |  |  |  |  |  |  |  |  |  |  |  |  |
| C-18 | -.002- | .833** | 0.152 | .969** | .984** | .997** | .993** | .992** | 0.269 | 1 |  |  |  |  |  |  |  |  |  |  |  |  |  |  |  |  |  |
| C-19 | 0.139 | .899** | 0.223 | .936** | .942** | .944** | .969** | .944** | .484* | .949** | 1 |  |  |  |  |  |  |  |  |  |  |  |  |  |  |  |  |
| C-20 | 0.002 | .830** | 0.139 | .972** | .986** | .997** | .992** | .993** | 0.253 | .999** | .944** | 1 |  |  |  |  |  |  |  |  |  |  |  |  |  |  |  |
| Pyr | 0.12 | .820** | 0.101 | .946** | .956** | .952** | .950** | .961** | 0.236 | .946** | .904** | .955** | 1 |  |  |  |  |  |  |  |  |  |  |  |  |  |  |
| Ace | 0.021 | .741** | 0.018 | .906** | .921** | .921** | .927** | .954** | 0.233 | .929** | .886** | .934** | .934** | 1 |  |  |  |  |  |  |  |  |  |  |  |  |  |
| Flu | -.046 | .795** | 0.112 | .953** | .970** | .984** | .976** | .993** | 0.227 | .989** | .923** | .993** | .952** | .942** | 1 |  |  |  |  |  |  |  |  |  |  |  |  |
| Ant | 0.059 | .842** | 0.123 | .979** | .990** | .991** | .988** | .985** | 0.246 | .988** | .947** | .990** | .964** | .949** | .975** | 1 |  |  |  |  |  |  |  |  |  |  |  |
| Phe | 0.115 | .813** | 0.107 | .943** | .953** | .950** | .948** | .933** | 0.238 | .943** | .920** | .938** | .905** | .895** | .901** | .966** | 1 |  |  |  |  |  |  |  |  |  |  |
| Chry | 0.16 | .715** | 0.047 | .798** | .798** | .765** | .773** | .771** | 0.210 | .748** | .740** | .766** | .839** | .776** | .769** | .793** | .708** | 1 |  |  |  |  |  |  |  |  |  |
| BaA | 0.059 | -.63** | -.179 | -.70** | -.72** | -.74** | -.73** | -.73** | -.180 | -.75** | -.68** | -.75** | -.64** | -.67** | -.75** | -.72** | -.66** | -.344 | 1 |  |  |  |  |  |  |  |  |
| BbF | 0.077 | 0.196 | -.207- | 0.307 | 0.304 | 0.272 | 0.256 | 0.272 | -.167 | 0.263 | 0.171 | 0.277 | 0.328 | 0.257 | 0.281 | 0.296 | 0.292 | 0.355 | -.349- | 1 |  |  |  |  |  |  |  |
| BkF | 0.097 | 0.078 | -.505* | 0.203 | 0.182 | 0.136 | 0.136 | 0.162 | -.202 | 0.128 | 0.037 | 0.147 | 0.219 | 0.217 | 0.158 | 0.19 | 0.191 | 0.289 | -.234- | .780** | 1 |  |  |  |  |  |  |
| BaP | 0.043 | .480* | -.186- | .616** | .625** | .606** | .629** | .666** | 0.147 | .610** | .603** | .630** | .748** | .765** | .654** | .669** | .573** | .750** | -.287- | 0.131 | 0.33 | 1 |  |  |  |  |  |
| Clay | -.160 | 0.114 | 0.026 | 0.237 | 0.244 | 0.279 | 0.253 | 0.278 | -.060 | 0.289 | 0.169 | 0.289 | 0.215 | 0.281 | 0.302 | 0.257 | 0.199 | 0.093 | -.270- | -.015 | -.140 | 0.13 | 1 |  |  |  |  |
| fine silt | -.169 | 0.072 | 0.039 | 0.172 | 0.179 | 0.221 | 0.197 | 0.206 | -.029 | 0.23 | 0.111 | 0.225 | 0.134 | 0.183 | 0.228 | 0.191 | 0.153 | 0.01 | -.271- | -.010- | -.165 | 0.011 | .961** | 1 |  |  |  |
| coarse silt | 0.146 | 0.395 | 0.387 | 0.344 | 0.355 | 0.374 | 0.378 | 0.330 | 0.135 | 0.369 | 0.303 | 0.363 | 0.302 | 0.224 | 0.342 | 0.337 | 0.316 | 0.163 | -.425-* | 0.311 | 0.099 | 0.018 | .473* | .534** | 1 |  |  |
| Sand | 0.053 | -.193 | -.167 | -.238 | -.244 | -.279 | -.268- | -.258- | -.076 | -.288- | -.183- | -.280- | -.185- | -.206- | -.276- | -.244- | -.211- | -.039- | 0.33 | -.087- | 0.121 | 0.022 | -.918** | -.961** | -.711** | 1 |  |
| gravel | 0.201 | -.066 | -.001 | -.199 | -.223 | -.250 | -.206- | -.221 | 0.349 | -.234- | -.133- | -.247- | -.258- | -.217- | -.248- | -.243- | -.192- | -.235- | 0.248 | -.086- | -.155 | -.336 | -.213- | -.207- | -.420-* | 0.158 | 1 |

| **. Correlation is significant at the 0.01 level (2-tailed). | | | | | | | | | | | | | | | | | | | | | | | | | | | | | | | | |
| --- | --- | --- | --- | --- | --- | --- | --- | --- | --- | --- | --- | --- | --- | --- | --- | --- | --- | --- | --- | --- | --- | --- | --- | --- | --- | --- | --- | --- | --- | --- | --- | --- |
| *. Correlation is significant at the 0.05 level (2-tailed). | | | | | | | | | | | | | | | | | | | | | | | | | | | | | | | | |
| c. Cannot be computed because at least one of the variables is constant. because DahA, Inp and BghiP is ND | | | | | | | | | | | | | | | | | | | | | | | | | | | | | | | | |
| N=23 |  |  |  |  |  |  |  |  |  |  |  |  |  |  |  |  |  |  |  |  |  |  |  |  |  |  |  |  |  |  |  |  |

Table S10. Correlation matrix between *n*-alkanes and PAHs at Nile Delta water.

|  | C9 | C10 | C11 | C12 | C13 | C14 | C15 | C16 | C17 | C18 | C19 | C20 | Ace | Flu | Ant | Phe | Chry | BaA | BbF | BkF | BaP |
| --- | --- | --- | --- | --- | --- | --- | --- | --- | --- | --- | --- | --- | --- | --- | --- | --- | --- | --- | --- | --- | --- |
| C9 | 1 |  |  |  |  |  |  |  |  |  |  |  |  |  |  |  |  |  |  |  |  |
| C10 | .535** | 1 |  |  |  |  |  |  |  |  |  |  |  |  |  |  |  |  |  |  |  |
| C11 | 0.048 | .661** | 1 |  |  |  |  |  |  |  |  |  |  |  |  |  |  |  |  |  |  |
| C12 | .468* | .693** | .710** | 1 |  |  |  |  |  |  |  |  |  |  |  |  |  |  |  |  |  |
| C13 | -.209 | 0.34 | .658** | 0.354 | 1 |  |  |  |  |  |  |  |  |  |  |  |  |  |  |  |  |
| C14 | 0.335 | .463* | .491* | .741** | -.114 | 1 |  |  |  |  |  |  |  |  |  |  |  |  |  |  |  |
| C15 | .444* | .613** | .432* | 0.339 | .472* | -.058 | 1 |  |  |  |  |  |  |  |  |  |  |  |  |  |  |
| C16 | .524** | .691** | .547** | .786** | 0.187 | .696** | .545** | 1 |  |  |  |  |  |  |  |  |  |  |  |  |  |
| C17 | .473* | .658** | .429* | .584** | 0.025 | .461* | .687** | .748** | 1 |  |  |  |  |  |  |  |  |  |  |  |  |
| C18 | .703** | .604** | 0.218 | .629** | -.138 | .604** | .491* | .893** | .751** | 1 |  |  |  |  |  |  |  |  |  |  |  |
| C19 | .763** | .486* | 0.007 | .510* | -.266 | .445* | .431* | .737** | .653** | .911** | 1 |  |  |  |  |  |  |  |  |  |  |
| C20 | .704** | .533** | 0.138 | .495* | -.224 | .511* | .511* | .846** | .729** | .939** | .909** | 1 |  |  |  |  |  |  |  |  |  |
| Ace | -.336- | 0.051 | 0.292 | 0.14 | 0.389 | 0.042 | 0.02 | 0.027 | 0.09 | -.125 | -.140 | -.168 | 1 |  |  |  |  |  |  |  |  |
| Flu | .726** | 0.22 | -.314 | -.015 | -.240 | -.168- | .521** | 0.264 | 0.345 | .514* | .648** | .582** | -.361 | 1 |  |  |  |  |  |  |  |
| Anth | -.008 | 0.243 | .561** | .433* | .423* | 0.398 | 0.231 | 0.268 | 0.248 | 0.133 | 0.01 | 0.044 | 0.362 | -.214 | 1 |  |  |  |  |  |  |
| Phe | -.114 | 0.206 | .604** | .457* | 0.384 | 0.394 | 0.105 | 0.271 | 0.131 | 0.06 | -.117 | -.009 | 0.032 | -.282 | .627** | 1 |  |  |  |  |  |
| Chry | -.083 | 0.102 | 0.257 | 0.278 | 0.166 | .407* | 0.003 | 0.245 | 0.097 | 0.122 | 0.003 | 0.141 | 0.067 | -.304 | .497* | 0.249 | 1 |  |  |  |  |
| BaA | -.261 | -.108- | 0.1 | 0.07 | 0.12 | 0.099 | -.047 | 0.183 | -.079 | 0.081 | -.080 | 0.023 | -.174 | -.229 | 0.138 | 0.214 | 0.303 | 1 |  |  |  |
| BbF | 0.155 | 0.171 | 0.144 | 0.211 | 0.096 | 0.138 | 0.058 | 0.219 | 0.105 | 0.113 | 0.13 | 0.181 | 0.197 | 0.207 | 0.099 | 0.074 | 0.105 | -.069 | 1 |  |  |
| BkF | 0.023 | 0.211 | .422* | .409* | 0.21 | 0.332 | -.029 | 0.234 | 0.113 | 0.108 | -.057 | 0.008 | -.076 | -.116 | 0.185 | .456* | 0.143 | -.092 | 0.376 | 1 |  |
| BaP | -.146 | -.059 | -.025 | -.050 | 0.219 | -.168 | -.138 | -.083 | -.254 | -.138 | -.100 | -.108 | 0.391 | -.034 | 0.1 | -.097 | 0.175 | -.025 | 0.182 | -.141 | 1 |

| **. Correlation is significant at the 0.01 level (2-tailed). | | | | | | | | | | | | | | | | | | | | | | | | | | | | | | | | |
| --- | --- | --- | --- | --- | --- | --- | --- | --- | --- | --- | --- | --- | --- | --- | --- | --- | --- | --- | --- | --- | --- | --- | --- | --- | --- | --- | --- | --- | --- | --- | --- | --- |
| *. Correlation is significant at the 0.05 level (2-tailed). | | | | | | | | | | | | | | | | | | | | | | | | | | | | | | | | |
| c. Cannot be computed because at least one of the variables is constant. because Pyr, DahA, Inp and BghiP is ND | | | | | | | | | | | | | | | | | | | | | | | | | | | | | | | | |
| N= 24 |  |  |  |  |  |  |  |  |  |  |  |  |  |  |  |  |  |  |  |  |  |  |  |  |  |  |  |  |  |  |  |  |

Table S11. Grain size analysis of the studied samples (%) [2,6].

| Compounds/  Stations | wt% clay | wt% fine silt | wt% coarse silt | wt% sand | wt% gravel | Total |
| --- | --- | --- | --- | --- | --- | --- |
| Ib | 0.51 | 3.07 | 1.88 | 90.86 | 3.66 | 99.98 |
| Ic | 8.52 | 41.62 | 35.33 | 14.49 | ND | 99.96 |
| IIa | 4.83 | 16.95 | 17.22 | 61.02 | ND | 100 |
| IIb | 5.07 | 17.40 | 22.66 | 54.9 | ND | 100 |
| IIc | 11.72 | 51.07 | 28.78 | 8.44 | ND | 100 |
| IIIa | 10.64 | 49.32 | 17.68 | 18.99 | 3.37 | 100 |
| IIIb | 7.14 | 31.17 | 32.23 | 29.48 | ND | 100 |
| IIIc | 11.74 | 57.65 | 21.66 | 7.07 | 1.88 | 100 |
| IVa | 11.32 | 45.74 | 13.18 | 29.73 | ND | 99.97 |
| IVb | 10.55 | 53.77 | 19.75 | 14.34 | 1.6 | 100 |
| IVc | 12.5 | 49.00 | 16.78 | 17.9 | 3.79 | 99.97 |
| Va | 0.58 | 1.80 | 2.13 | 95.49 | ND | 100 |
| Vb | 7.06 | 30.76 | 15.26 | 35.5 | 11.42 | 100 |
| Vc | 0.27 | 1.98 | 0.97 | 91.26 | 5.46 | 99.94 |
| VIa | 1.80 | 5.77 | 21.57 | 65.34 | 5.5 | 99.98 |
| VIb | 4.02 | 23.94 | 18.17 | 40.27 | 13.54 | 99.94 |
| VIc | 6.05 | 25.24 | 7.03 | 45.97 | 15.73 | 100 |
| VIIa | 2.60 | 8.20 | 24.88 | 64.35 | ND | 100 |
| VIIb | 9.71 | 53.57 | 30.51 | 6.25 | ND | 100 |
| VIIc | 10.57 | 60.75 | 25.13 | 3.56 | ND | 100 |
| VIIIa | 5.67 | 26.72 | 28.46 | 39.13 | ND | 99.98 |
| VIIIb | 8.01 | 45.96 | 31.54 | 11.86 | 2.62 | 99.99 |
| VIIIc | 7.14 | 40.31 | 33.6 | 17.63 | 1.29 | 99.97 |

wt.% clay: <2 microns; wt.% fine silt: 2 to 20 microns; wt% coarse silt: 20-63 microns; wt.% sand: 2-0.063 mm; wt.% gravel: >2 mm.

References

1. El Nemr, A., Moneer, A.A., Ragab, S. and El Sikaily, A., 2016. Distribution and sources of n-alkanes and polycyclic aromatic hydrocarbons in shellfish of the Egyptian Red Sea coast. Egypt. J. Aquat. Res. 42, 121-131.

2. Seopela, M.P., McCrindle, R.I., Combrinck, S., Augustyn, W., 2020. Occurrence, distribution, spatio-temporal variability and source identification of n-alkanes and polycyclic aromatic hydrocarbons in water and sediment from Loskop dam, South Africa. Water Res. 186, 116350.

3. USEPA 2011: United States Environmental Protection Agency; 0 = not carcinogenic, ++, +++ = strongly carcinogenic, petro (petrogenic); B2 Probable carcinogen, D - not classifiable as to human carcinogenicity (USEPA carcinogenic classification 2012), S.D. – standard deviation. ND – not detected, ERL - toxic effects range low, ERM - toxic effects range medium and TEF- Toxicity fator. Car. = Carcinogenicity; CP = Carcinogenic potency

4. USEPA (Environmental Protection Agency), 2012. Sediment contamination. <http://www.epa.gov/emap/maia/html/docs/Est5.pdfN>

5. United States Environmental Protection Agency (USEPA), 1993. Provisional Guidance for Quantitative Risk Assessment of Polycyclic Aromatic Hydrocarbons. EPA/600/R-93/089, Office of Research and Development, Washington DC, USA.

6. Hassaan, M.A. and El Nemr, A., 2021. Classification and identification of different minerals in the Mediterranean sediments using PSA, FTIR, and XRD techniques. Mar. Pollut. Bull. 173, p.113070. <https://doi.org/10.1016/j.marpolbul.2021.113070>
